# Toward a clinical diagnostic pipeline for *SPINK1* intronic variants

**DOI:** 10.1186/s40246-019-0193-7

**Published:** 2019-02-12

**Authors:** Xin-Ying Tang, Jin-Huan Lin, Wen-Bin Zou, Emmanuelle Masson, Arnaud Boulling, Shun-Jiang Deng, David N. Cooper, Zhuan Liao, Claude Férec, Zhao-Shen Li, Jian-Min Chen

**Affiliations:** 1 Department of Gastroenterology, Changhai Hospital, The Second Military Medical University, Shanghai, China; 2 Shanghai Institute of Pancreatic Diseases, Shanghai, China; 3 EFS, Univ Brest, Inserm, UMR 1078, GGB, 29200 Brest, France; 4 CHU Brest, Service de Génétique, Brest, France; 5 Institute of Medical Genetics, School of Medicine, Cardiff University, Cardiff, UK

**Keywords:** Aberrant splicing, Alamut software suite, Chronic pancreatitis, Cryptic splice site, Deep intronic variants, genomAD, In silico splicing prediction, Missing heritability, *SPINK1* gene, Splice site consensus sequence

## Abstract

**Background:**

The clinical significance of *SPINK1* intronic variants in chronic pancreatitis has been previously assessed by various approaches including a cell culture-based full-length gene assay. A close correlation between the results of this assay and in silico splicing prediction was apparent. However, until now, a clinical diagnostic pipeline specifically designed to classify *SPINK1* intronic variants accurately and efficiently has been lacking. Herein, we present just such a pipeline and explore its efficacy and potential utility in potentiating the classification of newly described *SPINK1* intronic variants.

**Results:**

We confirm a close correlation between in silico splicing prediction and results from the cell culture-based full-length gene assay in the context of three recently reported pathogenic *SPINK1* intronic variants. We then integrated in silico splicing prediction and the full-length gene assay into a stepwise approach and tested its utility in the classification of two novel datasets of *SPINK1* intronic variants. The first dataset comprised 16 deep intronic variants identified in 52 genetically unexplained Chinese chronic pancreatitis patients by sequencing the entire intronic sequence of the *SPINK1* gene. The second dataset comprised five novel rare proximal intronic variants identified through the routine analysis of the *SPINK1* gene in French pancreatitis patients. Employing a minor allele frequency of > 5% as a population frequency filter, 6 of the 16 deep intronic variants were immediately classified as benign. In silico prediction of the remaining ten deep intronic variants and the five rare proximal intronic variants with respect to their likely impact on splice site selection suggested that only one proximal intronic variant, c.194 + 5G > A, was likely to be of functional significance. Employing the cell culture-based full-length gene assay, we functionally analyzed c.194 + 5G > A, together with seven predicted non-functional variants, thereby validating their predicted effects on splicing in all cases.

**Conclusions:**

We demonstrated the accuracy and efficiency of in silico prediction in combination with the cell culture-based full-length gene assay for the classification of *SPINK1* intronic variants. Based upon these findings, we propose an operational pipeline for classifying *SPINK1* intronic variants in the clinical diagnostic setting.

**Electronic supplementary material:**

The online version of this article (10.1186/s40246-019-0193-7) contains supplementary material, which is available to authorized users.

## Background

Chronic pancreatitis has traditionally been defined as a chronic inflammatory process of the pancreas that leads to progressive and irreversible impairment of both exocrine and endocrine functions, with a focus on morphological changes. More recently, the disease has been redefined as a “pathologic fibro-inflammatory syndrome of the pancreas in individuals with genetic, environmental and/or other risk factors who develop persistent pathologic responses to parenchymal injury or stress”, with a focus on underlying pathogenic mechanisms [1]. In particular, genetic studies over the past two decades have underscored the importance of a trypsin-dependent pathway in the etiology of the disease [2–5]. One of the most extensively studied pancreatitis susceptibility genes, *SPINK1* (encoding pancreatic secretory trypsin inhibitor; MIM# 167790), is characterized by a diverse range of reported variants from point mutations to whole gene deletions (for a complete list, see ref. [6]). Pathogenic *SPINK1* variants predispose to pancreatitis by lowering the inhibitory capacity of prematurely activated trypsin within the pancreas. The clinical relevance of canonical splice site variants, nonsense mutations, or large-scale genomic deletions in the *SPINK1* gene is generally clear. By contrast, the clinical relevance of *SPINK1* promoter and enhancer variants [7–9], missense variants [10, 11], or intronic variants occurring outwith the canonical splice sites [12, 13] has often had to be ascertained by in vitro functional analysis.

In silico splicing prediction programs have been widely used to evaluate the functional effects of intronic variants in clinical genetics, either on their own or in combination with an in vitro splicing assay [14, 15]. In this regard, we have previously employed a cell culture-based full-length gene assay to systematically assess the functional impact of a series of *SPINK1* intronic variants [12, 13] and, more recently, we have noted a close correlation between the results from this assay and in silico splicing predictions [16]. The full-length gene assay has at least two advantages over the commonly used minigene splicing assay. First, the full-length gene assay preserves better the natural genomic context of the studied variants, a point of key importance given the highly context-dependent nature of splicing regulation [17]. Second, the full-length gene splicing assay can be readily used to evaluate intronic variants located near the first exon or last exon of the gene; by contrast, special adaptation of the minigene would normally be required for such variants to be analyzed, as exemplified by a recent publication [18].

*SPINK1* intronic variants continue to be reported in the literature [19–22] and additional *SPINK1* intronic variants, including those located in deep intronic regions, are certainly likely to emerge with the application of *high*-*throughput whole*-*genome sequencing* [23]*.* To rise to this challenge, establishment of a clinical diagnostic pipeline for the classification of *SPINK1* intronic variants is required. The aim of the present study was to develop such a pipeline and assess its efficacy and utility. To this end, we further explored the correlation of in silico splicing prediction and our cell-based full-length gene assay in the context of three recently reported pathogenic *SPINK1* variants. Then we integrated both the in silico splicing prediction procedure and the full-length gene assay into a stepwise approach in order to classify a series of *SPINK1* intronic variants newly discovered in Chinese and French pancreatitis patients.

## Results and discussion

### Further correlation of in silico splicing prediction and functional assay data in the context of three recently reported *SPINK1* splice site variants

Before going into the detail of the current study, we would like to make two points. The first refers to the experimental evaluation of the functional effect of intronic variants. Ideally, the disease-affected tissue/cells or surrogate tissue/cells that also highly express the gene of interest from the patients should be analyzed whenever possible. *SPINK1* mRNA is most abundantly expressed in the pancreas, with a median transcripts per kilobase million (TPM) of 4361 in accordance with the Genotype-Tissue Expression (GTEx) dataset [24]. Stomach ranks second for *SPINK1* mRNA expression, although the corresponding TPM is only 285 [24]. Neither tissue, but particularly the pancreas, is accessible in practice in terms of biopsy samples. The next and most commonly used strategy is to perform a splicing assay in a transient expression system, in which human cell lines of pathophysiological relevance should be employed whenever possible owing to the tissue specificity of the splicing process in some instances (see [25] and references therein). Unfortunately, no human pancreatic acinar cell lines are currently available. In the present study, we used human embryonic kidney 293 T (HEK293T) cells for the splicing assay as previously described [12, 13, 16]. It is possible that splicing in HEK293T cells may not always reflect the in vivo situation, a general drawback of splicing assay that employ non-pathophysiologically relevant cells [26].

The second point refers to in silico prediction of the impact of intronic variants on splicing. Of particular relevance, we have previously observed a close correlation of results from our full-length gene assay with those from the in silico splicing predictions in the context of 24 *SPINK1* intronic variants [16]. Findings pertaining to two variants merit especial attention. First, c.194 + 2T > C (or IVS3 + 2T > C) was shown to retain partial ability to generate wild-type transcripts by reverse transcription-polymerase chain reaction (RT-PCR) analysis of patient-derived stomach tissue [27] and also by our full-length gene assay [12]. It was predicted to be associated with only an ~ 12% decrease in the score for SpliceSiteFinder-like (wild-type score of 82.6 vs mutant score of 72.3) but invariably a score of 0 for MaxEntScan, NNSPLICE, and Human Splicing Finder [16]. Both predictions were correct, depending on one’s viewpoint. Thus, the prediction of SpliceSiteFinder-like was correct from the standpoint that c.194 + 2T > C was able to generate an appreciable level of wild-type transcript; the predictions of MaxEntScan, NNSPLICE, and Human Splicing Finder were correct from the standpoint that c.194 + 2T > C resulted in a significantly reduced level of normally spliced *SPINK1* transcript as compared to that of the wild-type allele. The second case involved the c.194 + 13T > G variant, which was predicted by different programs to generate a new and viable donor splice site but resulted in the generation of a trace amount of aberrantly spliced transcripts that was only detectable using specially designed allele-specific primers [16]. With hindsight, these two particular cases might reflect a limitation in splicing prediction programs analogous to that seen in missense variant prediction programs: “The main issue lies within the binary output of most models, which predict whether or not a variant has an effect but not its magnitude” [28]. Nonetheless, at least in the context of the 24 *SPINK1* intronic variants analyzed [16], the in silico prediction tools were collectively not found to yield false negative findings.

Bearing in mind the aforementioned considerations, we decided to further explore the cross-correlation of in silico predictions and our full-length gene splicing assay in the context of three recently reported *SPINK1* splice site variants, c.55 + 1G > A [19], c.194 + 1G > A [20], and c.88-1G > A [22]. These three variants are of unequivocally clinical significance by virtue of their disruption of splice site consensus sequences (Additional file 1: Figure S1), as predicted by SpliceSiteFinder-like, MaxEntScan, NNSPLICE, and GeneSplicer, made available via the Alamut software suite, under default conditions [29].

**Fig. 1:**
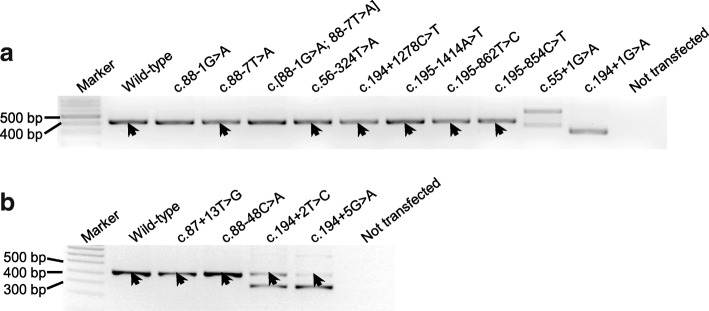
Results from the cell culture-based full-length gene assay. **a**, **b** RT-PCR analyses of HEK293T cells transfected with full-length *SPINK1* gene expression constructs carrying respectively the wild-type and indicated intronic variants. Normal transcripts (confirmed by sequencing) are indicated by arrows. In **b**, the lower bands generated by c.194 + 2 T > C and c.194 + 5G > A were found to be identical by sequencing, with exon 3 being skipped. See Fig. 2 for the precise splicing outcomes of the three recently reported *SPINK1* splice site variants

**Fig. 2:**
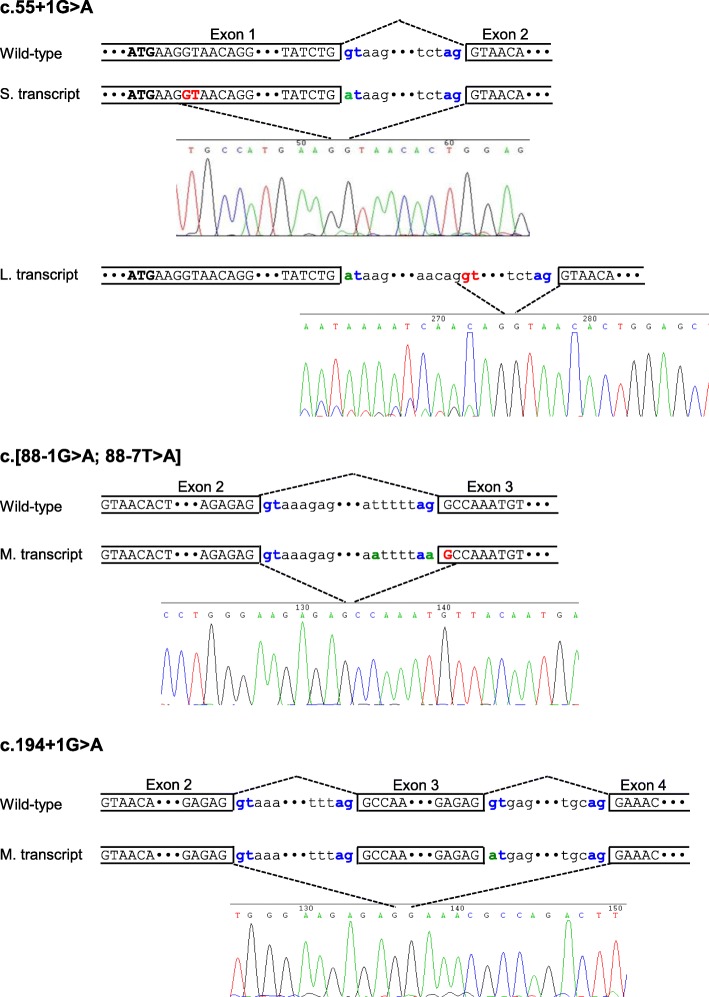
Splicing outcomes of the three recently reported *SPINK1* splice site variants as determined by the cell culture-based full-length gene assay. Normal splicing in the context of the wild-type sequence and aberrant splicing in the context of the mutant sequence are illustrated for each of the three variants. The splice donor signal (GT) and splice acceptor signal (AG) are highlighted in blue. Variants occurred within the splice sites are highlighted in green. In c.55 + 1G > A, the two novel splice donor sites used for aberrant splicing are highlighted in red. In c.[88-1G > A; 88-7 T > A], the c.88-1G > A variant shifted the AG site by one base, resulting in the skipping of the first nucleotide of exon 3 (i.e., the G highlighted in red). *S* shorter, *L* longer, *M* mutant

We then characterized the splicing defects associated with the three splice site variants by means of our previously established cell culture-based full-length gene assay [12, 13]. RT-PCR analysis of HEK293T cells transfected with the full-length *SPINK1* gene construct harboring the c.55 + 1G > A variant showed two aberrant transcripts (Fig. 1a). Subsequent sequencing revealed that the shorter transcript resulted from the activation of a cryptic splice donor site within exon 1 (at position c.7_8), resulting in the deletion of the 3′ end of exon 1; the longer transcript resulted from activation of a cryptic splice acceptor site within intron 1 (at position c.55 + 141_55 + 142), resulting in the insertion of the 5′ end of intron 1 into the transcript (Fig. 2).

The c.194 + 1G > A variant was found to result in the generation of a single aberrant transcript (Fig. 1a), in which exon 3 was skipped (Fig. 2).

The c.88-1G > A variant [22] was found to be *in cis* with c.88-7 T > A, located only five bases away (Additional file 1: Figure S2). Given that neither of these two variants is present in the Genome Aggregation Database (genomAD) [30], it is possible that they were generated simultaneously as a single mutational event [31, 32]. Irrespective of whether or not the two variants were generated simultaneously, they should be named c.[88-1G > A; 88-7 T > A] in accordance with the Human Genome Variation Society (HGVS) recommendations [33].

The c.[88-1G > A; 88-7 T > A] variant is very likely to affect splicing due to the c.88-1G > A component (Additional file 1: Figure S1). However, the splicing outcome of the c.88-1G > A variant may be modified by the juxtaposition of the c.88-7 T > A variant, even although the latter on its own was predicted not to significantly affect splice site selection (Additional file 1: Figure S3). To explore this possibility, we compared the splicing outcomes in vitro of c.88-1G > A alone, c.88-7 T > A alone, and c.[88-1G > A; 88-7 T > A]. RT-PCR analyses of HEK293T cells transfected with the corresponding full-length gene constructs invariably generated a single band of similar size to that of the wild-type (Fig. 1a). Subsequent sequencing revealed that c.88-1G > A alone and c.[88-1G > A; 88-7 T > A] alone resulted in identical skipping of the first nucleotide of exon 3 (i.e., the splice site was shifted by one nucleotide; Fig. 2) of the *SPINK1* gene while c.88-7 T > A alone generated only wild-type transcripts. Consequently, it may be concluded that the functional effect of c.[88-1G > A; 88-7 T > A] was conferred solely by the c.88-1G > A variant.

Taken together, we have provided further evidence for a good correlation between in silico splicing predictions and our functional assay of *SPINK1* intronic variants. Indeed, our cell culture-based full-length gene assay not only validated the predicted impact on splicing but also elucidated the precise mRNA splicing consequences of specific pathogenic variants. The latter is key to understanding the genotype-phenotype relationship since aberrantly spliced transcripts may not invariably lead to the synthesis of proteins characterized by complete loss-of-function.

### Integration of splicing prediction and functional assay into a stepwise procedure for classifying newly found *SPINK1* variants

Data directly comparing the incidences and clinical features of chronic pancreatitis between Chinese and European populations are lacking. By contrast, marked ethnic differences were noted between them in terms of genetic predisposition to the disease, exemplified by three recent findings: the *CEL*-*HYB* risk allele [34] was found to be absent in the Chinese population [35]; rare functional *CPA1* variants [36] were not enriched in Chinese chronic pancreatitis patients [37]; and the common *CTRB1*-*CTRB2* inversion allele [38] did not contribute to disease risk in the Chinese population due to near allele fixation [39]. Significant differences also exist between Chinese and European populations in terms of the spectrum and frequency of variants in each of the four firmly established pancreatitis susceptibility genes (i.e., *SPINK1* [40], *PRSS1* [41], *CTRC* [42, 43], and *CFTR* [44, 45]) [22].

A comprehensive analysis of the *SPINK1*, *PRSS1*, *CTRC*, and *CFTR* genes in 253 young French chronic pancreatitis revealed that ~ 52% of the studied patients remained genetically unexplained [46]. Remarkably, the proportion of Chinese patients that remained genetically unexplained after mutational analysis of the above four genes (i.e., ~ 50%) [22] is quite comparable to that in the French patients. As part of our attempt to identify the “missing heritability,” we performed targeted resequencing of the deep intronic sequence of the *SPINK1* gene in 52 genetically unexplained Chinese chronic pancreatitis patients using previously described methods [13]. [Note that the proximal intronic regions of the *SPINK1* gene had previously been analyzed [22].] This resulted in the identification of 16 deep *SPINK1* intronic variants (Table 1). In addition, during the routine analysis of the *SPINK1* gene (focusing on coding and proximal intronic sequences) in French pancreatitis patients, we identified five rare proximal *SPINK1* intronic variants that had not previously been described in the literature (Table 2). In the five respective French carriers, no known disease-causing variants in the *PRSS1* gene were found but other pancreatitis susceptibility genes remain to be analyzed. This does not affect the conclusion of the present study in any way.

**Table 1:** *SPINK1* deep intronic variants found in the 52 Chinese patients with chronic pancreatitis

Intron	Variant		No. of carriers^a^	Allele frequency in patients	Allele frequency in the East Asian population^b^	rs number	In silico prediction^c^	In vitro validation
	cDNA numbering	gDNA numbering (chr 5, hg19)						
Minor allele frequency of ≥ 5% (all have previously been described [3])								
2	c.88-352A > G	g.147208043 T > C	51 (37 hom.)	0.846	0.819^d^	rs6580502	No effect	Not done
3	c.194 + 1159C > G	g.147206426G > C	10	0.096	0.070	rs1897577	No effect	Not done
3	c.195-1645G > C	g.147205914C > G	43 (17 hom.)	0.577	0.571^d^	rs17717320	No effect	Not done
3	c.195-1570C > A	g.147205839G > T	44 (22 hom.)	0.635	0.619^d^	rs17703305	No effect	Not done
3	c.195-478 T > G	g.147204747A > C	10	0.096	0.070	rs17774073	No effect	Not done
3	c.195-323C > T	g.147204592G > A	44 (21 hom.)	0.625	0.618^d^	rs4705202	No effect	Not done
Minor allele frequency of < 5% (all have not previously been described [3])								
1	c.56-609G > C	g.147209802C > G	1	0.00962	Absent from genomAD	Not available	No effect	Not done
1	c.56-324 T > A	g.147209517A > T	2	0.01923	0.00963	rs546549375	No effect	Yes
3	c.194 + 671C > T	g.147206914G > A	1	0.00962	0.00193	rs889082209	No effect	Not done
3	c.194 + 723C > T	g.147206862G > A	1	0.00962	0.00193	rs573757839	No effect	Not done
3	c.194 + 855G > A	g.147206730C > T	1	0.00962	0.00705	rs543534355	No effect	Not done
3	c.194 + 1278C > T	g.147206307G > A	3	0.02885	0.01797	rs118005432	No effect	Yes
3	c.194 + 1599G > A	g.147205986C > T	1	0.00962	Absent from genomAD	Not available	No effect	Not done
3	c.195-1414 T>A^e^	g.147205683A>T^e^	1	0.00962	0.00000^f^	rs2436411^e^	No effect	Yes
3	c.195-862 T > C	g.147205131A > G	1^g^	0.01923	Absent from genomAD	Not available	No effect	Yes
3	c.195-854C > T	g.147205123G > A	1	0.00962	0.00062	rs1055746254	No effect	Yes

^a^Number of homozygotes (hom.) is indicated in parentheses wherever applicable

^b^Data are in accordance with genomAD (as of October 31, 2018)

^c^Effect on spice site selection predicted by SpliceSiteFinder-like, MaxEntScan, NNSPLICE and GeneSplicer under default conditions

^d^The alternative minor allele frequency is of ≥ 5%

^e^In hg19, the reference sequence at this position is the minor allele sequence

^f^Absent in the East Asian population but present in other population(s)

^g^The carrier is a homozygote. The presence of a large deletion spanning the position of interest cannot be excluded

**Table 2:** Rare proximal *SPINK1* intronic variants found in French pancreatitis patients

Region	Variant^a^		Allele frequency in the European (non-Finnish) population^b^	rs number	In silico prediction^c^	In vitro validation
	cDNA numbering	gDNA numbering (chr5, hg19)				
Intron 2	c.87 + 13 T > G	g.147209149A > C	Absent from genomAD	Not available	No effect	Yes
Intron 2	c.88-48C > A	g.147207739G > T	0.00001	rs753830042	No effect	Yes
Intron 3	c.194 + 5G > A	g.147207580C > T	Absent from genomAD	Not available	Significantly reduced the score for the c.194 + 2 splice site	Yes
Intron 3	c.194 + 32 T > C	g.147207553A > G	0.00000^d^	rs770552173	No effect	Not done
Intron 3	c.195-21 T > A	g.147204290A > T	0.00014	rs377350168	No effect	Not done

^a^Each variant was found once in some 4000 patients subjected to routine analysis of the *SPINK1* gene. None of the variants have been described in the Genetic Risk Factors in Chronic Pancreatitis Database [6] (as of October 29, 2018)

^b^Data in accordance with genomAD (as of October 31, 2018)

^c^Effect on splice site selection as predicted by SpliceSiteFinder-like, MaxEntScan, NNSPLICE and GeneSplicer under default conditions

^d^Absent in the European (non-Finnish) population but present in other population(s)

In the following sections, we describe how we attempted to integrate in silico splicing predictions and our full-length gene assay into a stepwise approach to classify the above two datasets of *SPINK1* intronic variants.

#### First step: population frequency filtering

Demonstrating the functionality of a given variant is a prerequisite for any claim of pathogenicity to be credible. A primary consideration when predicting whether a variant is likely to have a functional effect is its population frequency [47]; the rarer the variant, the more likely it is to exert a pathogenic effect. A minor allele frequency (MAF) of > 1% in the control population is the most frequently used threshold for defining “common” variants. Here, we employed a conservative threshold, a MAF of > 5%, for population frequency filtering, using data from genomAD [30] as a reference. Using allele frequency in the East Asian population as a filter would have resulted in 6 of the 16 deep intronic variants found in the Chinese patients being classified as benign (Table 1). Indeed, all six of these common variants have previously been described and annotated as benign in the Genetic Risk Factors in Chronic Pancreatitis Database [6]. It should be noted that in the case of four of the six common variants, it is the minor allele that is used as the reference sequence. We did not attempt to convert the corresponding major allele frequencies to the alternative MAFs in these cases (Table 1).

#### Second step: in silico prediction on splice site selection

**Fig. 3:**
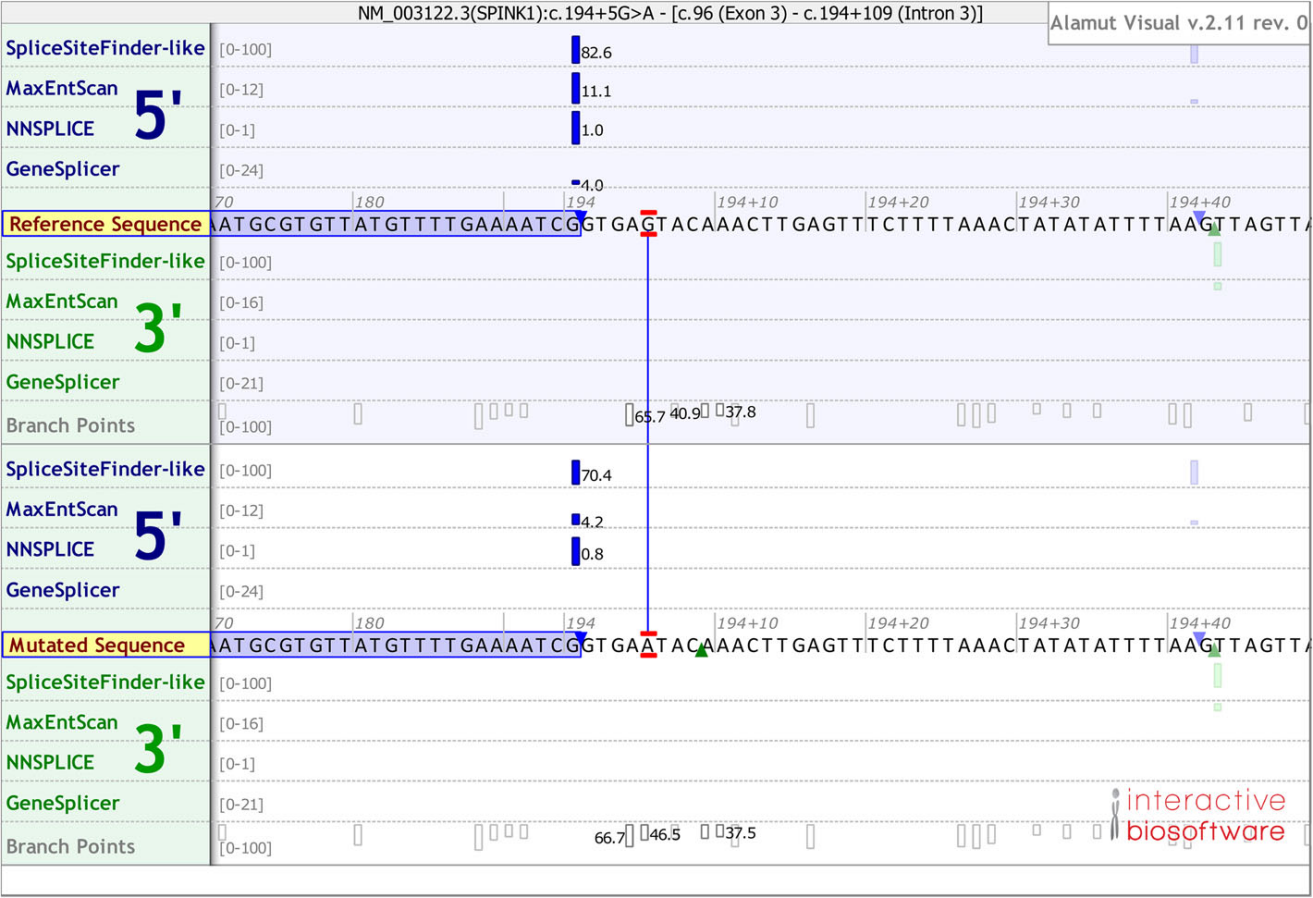
Splicing effect of the proximal c.194 + 5G > A variant as predicted by the Alamut software suite

None of the remaining ten deep *SPINK1* intronic variants, all of which had an allele frequency of < 5% in the East Asian population (Table 1), have been previously described in the Genetic Risk Factors in Chronic Pancreatitis Database [6]. These variants, together with the five rare proximal variants found in the French patients (Table 2), were subjected to in silico splicing prediction (i.e., disruption of known splice sites or creation of novel splice sites were sought) by means of SpliceSiteFinder-like, MaxEntScan, NNSPLICE, and GeneSplicer made available via the Alamut software suite, under default conditions [29]. However, only the proximal c.194 + 5G > A variant was predicted to be of functional significance by virtue of it significantly reducing the splice site consensus scores (defined here as a reduction of ≥ 10% of the wild-type value across all four prediction programs) as compared to the wild-type allele (Fig. 3). By contrast, none of the ten rare deep intronic variants found in Chinese patients or the other four rare proximal variants found in French patients were predicted to significantly reduce the splice site consensus scores or generate a novel splice site (Additional file 1: Figures S4 and S5). Additionally, we performed the same predictions for the six common deep *SPINK1* intronic variants (Table 1) but none were predicted to have a functional effect (Additional file 1: Figure S6). In short, of the 16 common and 5 rare *SPINK1* intronic variants, the proximal c.194 + 5G > A variant was the only one predicted to be of functional significance (Tables 1 and 2).

#### Third step: functional validation

We performed functional analysis of the predicted functionally significant c.194 + 5G > A variant by means of our cell culture-based full-length gene assay. We also included, as negative controls, seven variants predicted to be non-functional (including both deep and proximal variants; Tables 1 and 2), as a means to cross-correlate in silico prediction and the results of our functional assay. RT-PCR analyses of the respectively transfected HEK293T cells confirmed the splicing predictions in all cases. Thus, a single transcript of similar size to the wild-type was observed in all seven predicted non-functional variants (i.e., c.56-324 T > A, c.194 + 1278C > T, c.195-1414 T > A, c.195-862 T > C, and c.195-854C > T in Fig. 1a; and c.87 + 13 T > G and c.88-48C > A in Fig. 1b); subsequent sequencing confirmed that all these transcripts were identical to the wild-type sequence. By contrast, c.194 + 5G > A generated a splicing pattern that was very similar to that of the pathogenic c.194 + 2 T > C variant [12], comprising a normally spliced band and an aberrantly spliced band (exon 3 skipped). It should be noted that the c.194 + 5G > A variant retained fewer normally spliced transcripts as compared to the c.194 + 2 T > C variant (Fig. 1b), an observation that argues for it being a novel pathogenic variant.

The precise splicing outcomes of pathogenic *SPINK1* intronic variants described to date are summarized in Table 3. All these pathogenic intronic variants are located either in the canonical splice sites or very close to the exon/intron junctions. This concurs with findings from many disease genes, probably for two reasons: splice-defining *cis*-acting sequence elements are predominantly located within proximal intronic regions [48] and the large size of the intronic regions renders routine screening impractical. In this regard, take two examples of recent large-scale analyses, one in the context of human cancer [49] and the other in the context of Stargardt disease [26]: none of the intronic variants under study were located within deep intronic regions. This notwithstanding, pathogenic variants do occur within deep intronic regions, and are often discovered by transcript analysis or whole-genome sequencing (e.g., [50–52]). In terms of their functional consequences, pathogenic variants in deep intronic regions often appear to be able to generate some wild-type transcripts (e.g., all three *USH2A* deep intronic variants reported in [50] were shown to do so in a minigene assay). In terms of their clinical consequences, pathogenic variants in deep intronic regions may be associated with a broad phenotypic spectrum, as exemplified by three *CFTR* deep intronic variants [52]. Pathogenic variants in the deep intronic regions of the *SPINK1* gene may be identified in the future, when whole-genome sequencing is routinely used for clinical diagnosis.

**Table 3:** Precise splicing outcomes of the pathogenic *SPINK1* intronic variants described to date

Region	Variant nomenclature^a^			Description on splicing mechanisms and outcomes	Reference pertaining to functional analysis
	At DNA level	At RNA level	At protein level		
Intron 1	c.55 + 1G > A	r.7_55del; and r.55_56ins55 + 1_55 + 140	p.Gly5Leufs*74; and p.Gly19Aspfs*11	Activation of a cryptic splice donor site within exon 1 (at position c.7_8), resulting in the deletion of the 3′ end of exon 1; and activation of a cryptic splice acceptor site within intron 1 (at position c.55 + 141_55 + 142), resulting in the insertion of the 5′ end of intron 1 into the transcript	This study
Intron 2	c.87 + 1G > A	r.56_87del	p.Asn20Glnfs*5	Exon 2 skipping	[12]
Intron 2	c.[88-1G > A; 88-7 T > A]	r.88del	p.Ala30Profs*65	Skipping of the first nucleotide of exon 3 (the functional effect was derived entirely from the c.88_1G > A component variant)	This study
Intron 3	c.194 + 1G > A	r.88_194del	p.Ala30Glufs*35	Complete exon 3 skipping	This study
Intron 3	c.194 + 2 T > C	r.88_194del	p.Ala30Glufs*35	Skipping of exon 3 in 90% of transcripts	[12]
Intron 3	c.194 + 5G > A	r.88_194del	p.Ala30Glufs*35	Almost complete exon 3 skipping	This study

^a^Variant nomenclature followed HGVS recommendations (http://www.hgvs.org/mutnomen/)

Finally, it is pertinent to mention that any *SPINK1* intronic variant that has been classified as benign may actually occur in *cis* with a functional variant located elsewhere in the coding sequence or regulatory regions of the gene. In order to explore this possibility, we searched all the currently studied *SPINK1* intronic variants with a known rs number (Tables 1 and 2) in the GTEx dataset [24]. Only three SNPs, rs6580502, rs17703305, and rs4705202, all of which have a MAF of > 5 in the general population, were associated with a reduced *SPINK1* expression; all the expression data were obtained from the lung tissue.

## Conclusions

In the context of three recently reported *SPINK1* splice site variants, we have provided further evidence for a close correlation between in silico splicing predictions and the results of our functional assay of *SPINK1* intronic variants. In the context of two new datasets of *SPINK1* intronic variants, we then demonstrated the accuracy and efficiency of in silico splicing prediction in combination with the cell culture-based full-length gene assay in variant classification. In so doing, we elucidated the precise splicing consequences of the three recently reported *SPINK1* splice site variants and identified and functionally characterized a novel pathogenic variant, c.194 + 5G > A. Based on the findings of this study and previous studies, we propose the following clinical diagnostic pipeline for classifying *SPINK1* intronic variants. The first step applies a population frequency filter using data in genomAD as a reference and employing a conservative MAF of ≥ 5% as a threshold. In the second step, the impact of the remaining rare variants on splice site selection is predicted. These two steps proved highly effective at classifying most of the detected *SPINK1* intronic variants as benign. Thus, in practice, only a very small number of *SPINK1* intronic variants (those predicted to affect splice site selection) actually needed to be functionally validated. We believe that the application of this procedure will greatly facilitate the classification of *SPINK1* intronic variants in a clinical diagnostic setting. This notwithstanding, it should be noted that the number of in-parallel tested *SPINK1* intronic variants is still relatively small. Consequently, we would recommend that functional analysis be employed once an intronic variant is suggested to be of functional significance by two or even one splicing prediction programs. Moreover, the threshold MAF for population frequency filtering (the reference population must be the same as the proband population) may be redefined as more data become available. Finally, it should be appreciated that an accurate determination of the pathogenic relevance of any *SPINK1* intronic variant in chronic pancreatitis is not only important from a mechanistic viewpoint [1] but also provides potential therapeutic targets as shown in other genes (e.g., [51, 53]).

## Methods

### Identification of *SPINK1* intronic variants in Chinese and French pancreatitis patients

Fifty-two Han Chinese chronic pancreatitis patients, whose age of disease onset was known to be ≤ 20 years or whose disease diagnosis was made at ≤ 20 years, participated this study. These patients, whose pancreatitis had remained genetically unexplained after mutational analysis of the entire coding regions and exon/intron boundaries of four pancreatitis susceptibility genes (i.e., *SPINK1* [40], *PRSS1* [41], *CTRC* [42, 43], and *CFTR* [44, 45]) [22], were searched for possible pancreatitis-predisposing variants occurring within deep *SPINK1* intronic regions in accordance with previously described procedures [13]. Proximal *SPINK1* intronic variants were identified through routine mutational screening of the entire coding region and exon/intron boundaries of the *SPINK1* gene in French pancreatitis patients by means of high-resolution DNA melting (HRM) analysis [54]. All *SPINK1* intronic variants were subjected to independent PCR amplification and Sanger sequencing. Informed consent was obtained from each participant. This study was approved by the respective Ethics Committees of Changhai Hospital in Shanghai and the University Hospital in Brest.

### Nomenclature of *SPINK1* intronic variants

Nomenclature for the description of *SPINK1* intronic sequence variants followed HGVS recommendations [33]. It should however be noted that whereas the *SPINK1* gene comprises five exons, in accordance with mRNA reference sequence accession NM_003122.3, the gene expressed in the exocrine pancreas comprises only four exons [55, 56]. It is the latter gene structure that is used by both pancreatitis genetics researchers [6, 9, 13, 40, 57] and Ensembl (refer to ENSG00000164266) [58]. The traditional IVS (InterVening Sequence; i.e., an intron) nomenclature for describing *SPINK1* intronic variants corresponds to the four-exon gene structure. In this study, in accordance with convention, we used the four-exon gene structure of pathophysiological relevance to define *SPINK1* intron numbers. Thus, in the current work, the *SPINK1* gene is regarded as harboring three introns. Since the first exon in accordance with mRNA reference sequence accession NM_003122.3 is non-coding, the variant nomenclature following HGVS recommendations is unaffected.

### Allele frequency reference

Data in genomAD [30] were used as a reference for population frequency filtering.

### In silico prediction of impact on splice site selection

In silico prediction of the impact of specific variants on splice site choice was performed using Alamut® Visual v.2.11 rev. 0 that included four prediction algorithms viz. SpliceSiteFinder-like, MaxEntScan, NNSPLICE and GeneSplicer under default conditions [29].

### Cell culture-based full-length gene assay

The wild-type expression vector harboring the full-length genomic *SPINK1* gene has been previously described [59]. It was used to generate the full-length expression constructs harboring respectively the selected *SPINK1* intronic variants by means of the QuikChange II XL Site-Directed Mutagenesis Kit (Agilent Technologies). In vitro mutagenesis, HEK293T cell culture, transfection, RT-PCR, and real-time quantitative RT-PCR analyses were performed essentially as previously described [12, 16].

## Additional files

Additional file 1: **Figure S1.** Alamut-predicted impact on splice site selection of the three recently reported *SPINK1* spice site variants. **Figure S2.** Presence of the c.88-1G > A (chr5:g.147207692C > T) in *cis* with a closely spaced variant, c.88-7 T > A (chr5:g.147207698A > T), in a Chinese patient with chronic pancreatitis. **Figure S3.** Alamut-predicted impact on splice site selection of the proximal c.88-7 T > A variant. **Figure S4.** Alamut-predicted impact on splice site selection of the 10 deep *SPINK1* intronic variants with a minor allele frequency of < 5% in the East Asian population. **Figure S5.** Alamut-predicted impact on splice site selection of the other four proximal *SPINK1* intronic variants found in the French pancreatitis patients. **Figure S6.** Alamut-predicted impact on splice site selection of the six deep *SPINK1* intronic variants with a minor allele frequency of ≥5% in the East Asian population. (PDF 2391 kb)

## Abbreviations

genomAD: The Genome Aggregation Database
GTEx: Genotype-tissue expression
MAF: Minor allele frequency
RT-PCR: Reverse transcription-polymerase chain reaction
TPM: Transcripts per kilobase million
