# Supplementary material for: Toward a clinical diagnostic pipeline for SPINK1 intronic variants
Source: Hum Genomics. 2019 Feb 12;13:8. doi: 10.1186/s40246-019-0193-7 (PMC6373104; doi:10.1186/s40246-019-0193-7)
Supplement: Supplementary file 1 — Figure S1. Alamut-predicted impact on splice site selection of the three recently reported SPINK1 spice site variants. Figure S2. Presence of the c.88-1G > A (chr5:g.147207692C > T) in cis with a closely spaced variant, c.88-7 T > A (chr5:g.147207698A > T), in a Chinese patient with chronic pancreatitis. Figure S3. Alamut-predicted impact on splice site selection of the proximal c.88-7 T > A variant. Figure S4. Alamut-predicted impact on splice site selection of the 10 deep SPINK1 intronic variants with a minor allele frequency of < 5% in the East Asian population. Figure S5. Alamut-predicted impact on splice site selection of the other four proximal SPINK1 intronic variants found in the French pancreatitis patients. Figure S6. Alamut-predicted impact on splice site selection of the six deep SPINK1 intronic variants with a minor allele frequency of ≥5% in the East Asian population. (PDF 2391 kb) [file 40246_2019_193_MOESM1_ESM.pdf]

c.55+1G>A

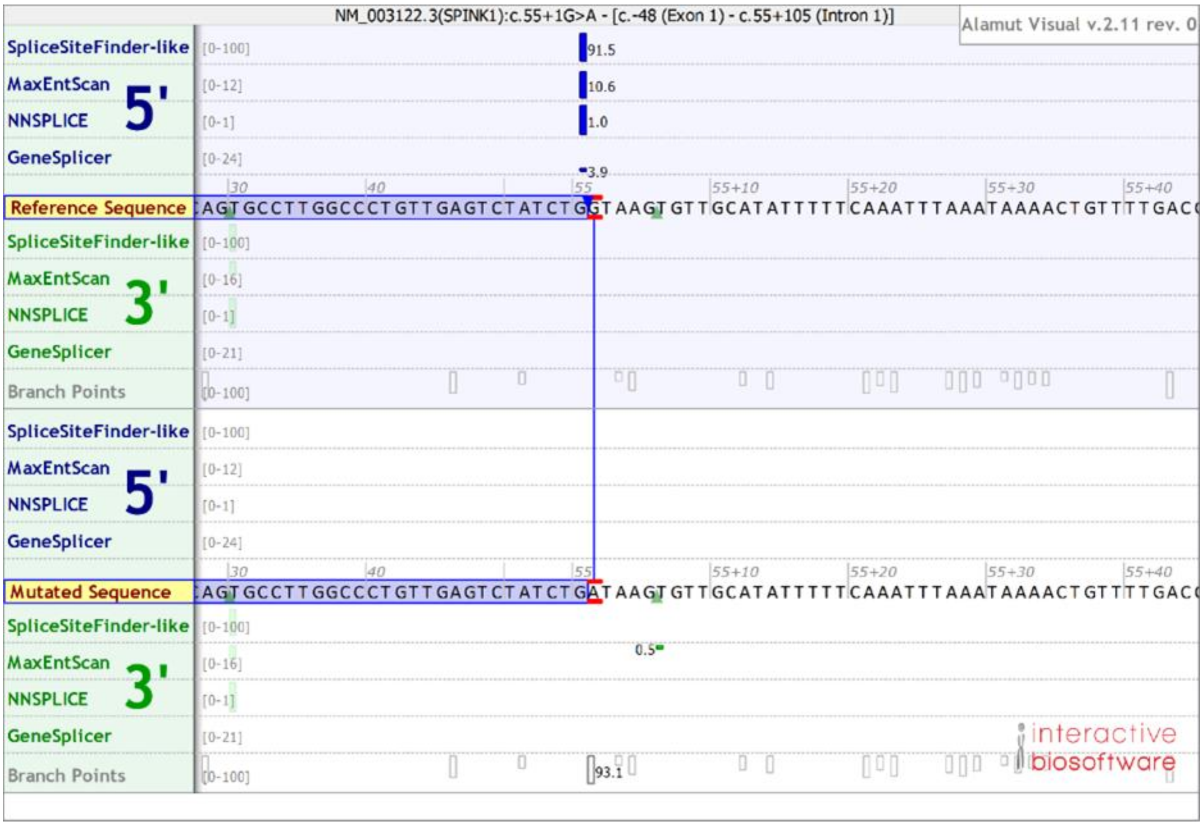

c.88-1G>A

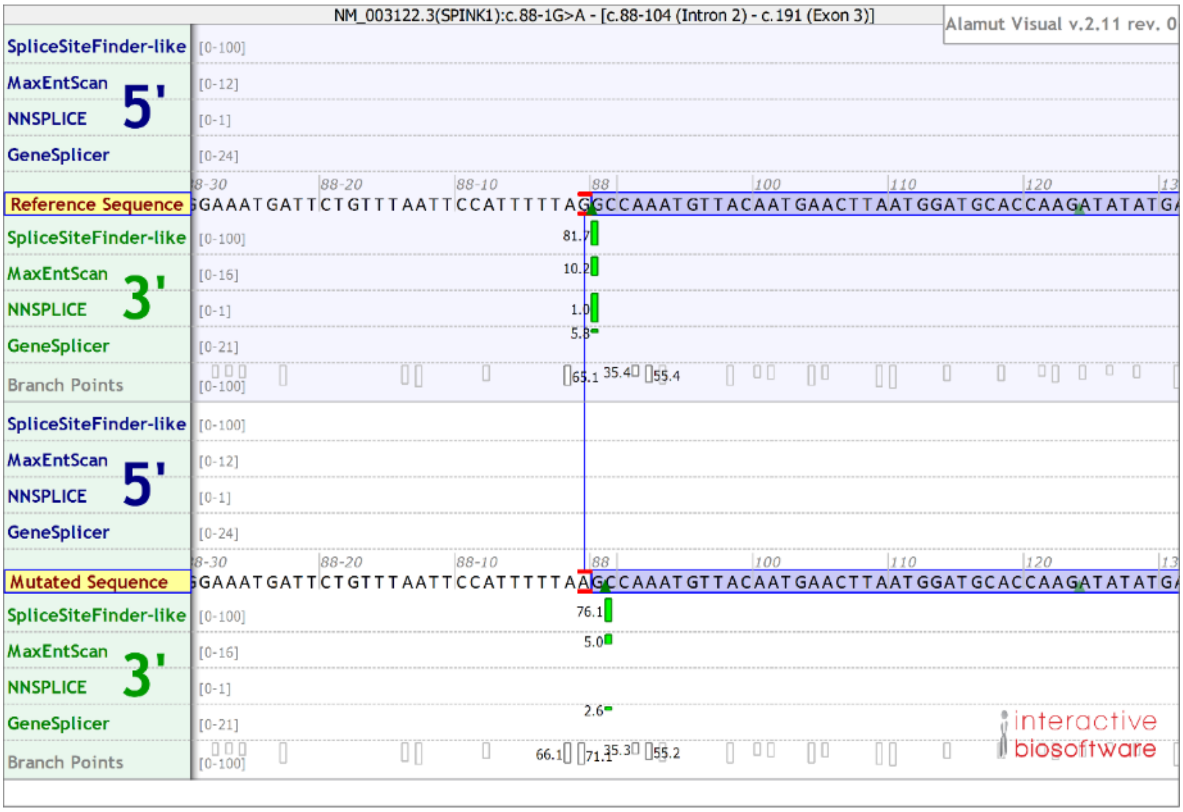

Figure S1. Alamut-predicted impact on splice site selection of the three recently reported *SPINK1* spice site variants.

c.194+1G>A

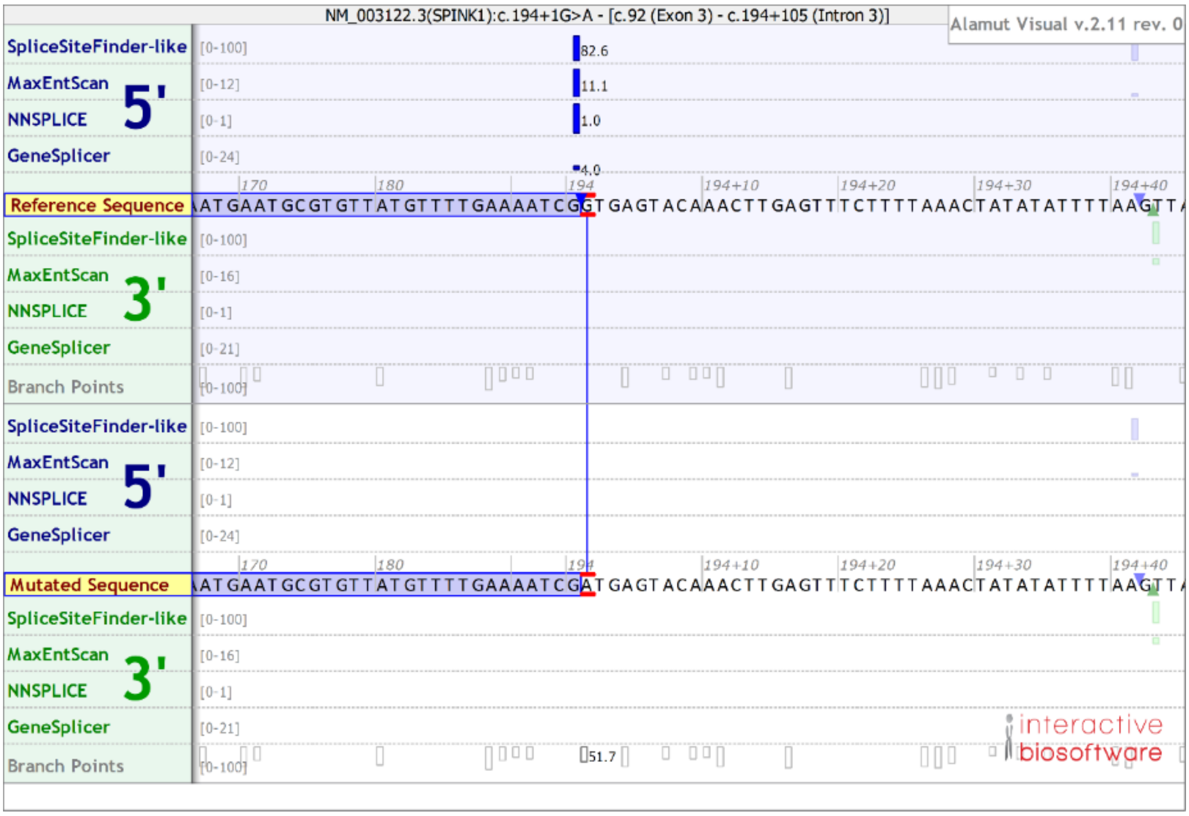

Figure S1 (continued)

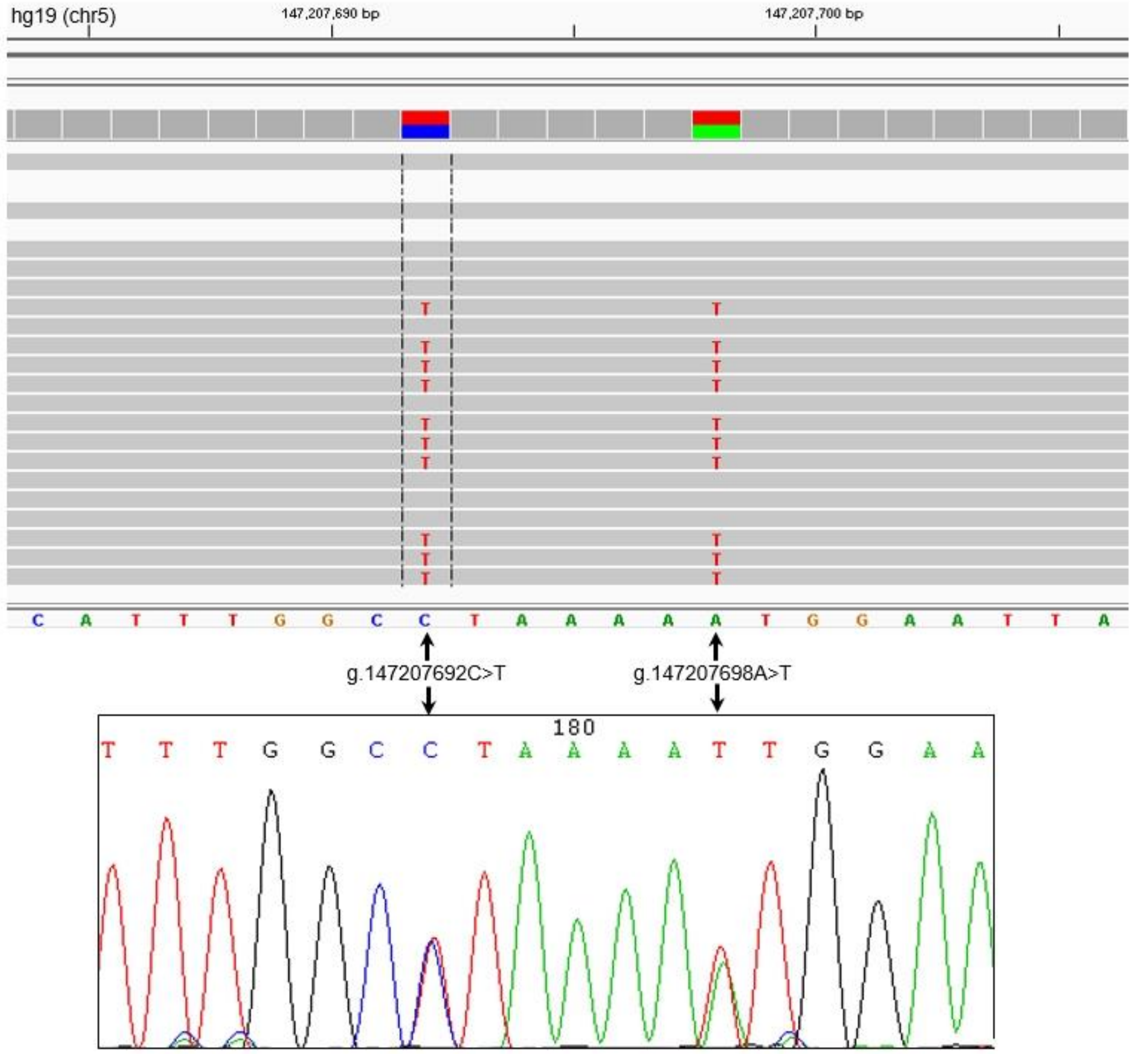

Figure S2. Presence of the c.88-1G>A (chr5:g.147207692C>T) in *cis* with a closely spaced variant, c.88-7T>A (chr5:g.147207698A>T), in a Chinese patient with chronic pancreatitis. Upper panel, the two variants identified by next-generation sequencing. Lower panel, presence of the two variants in the carrier confirmed by Sanger sequencing.

c.88-7T>A

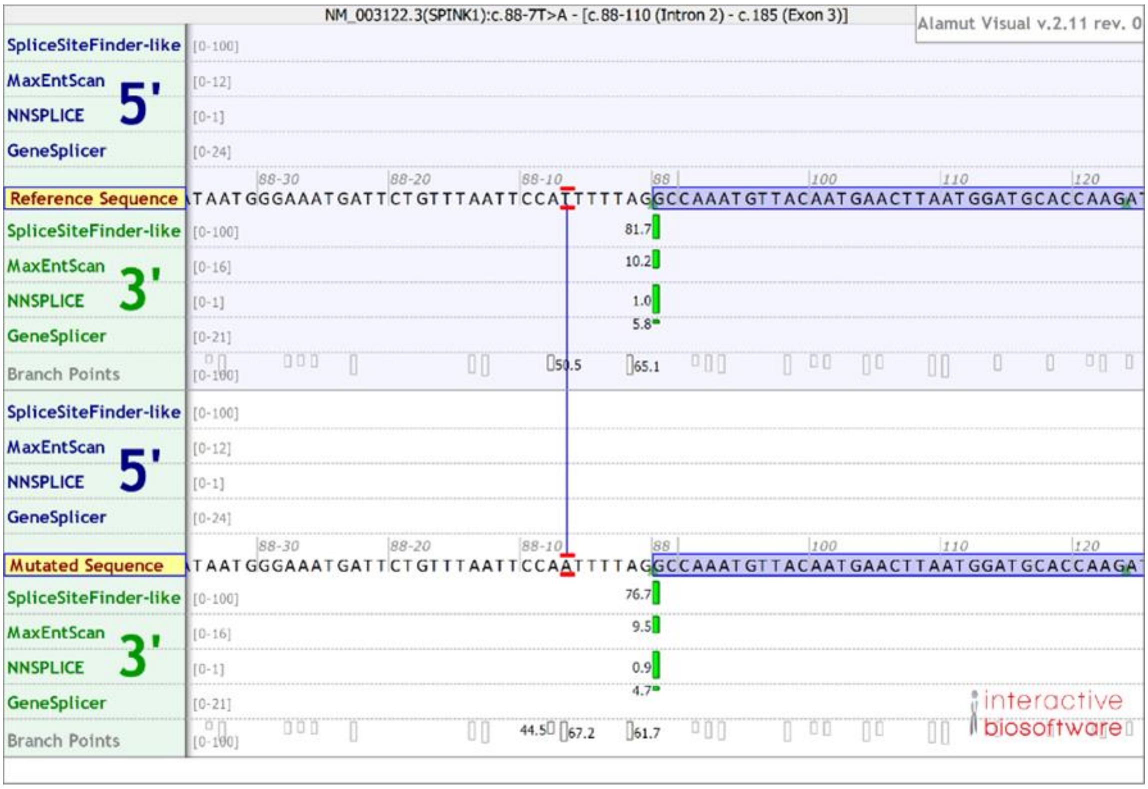

Figure S3. Alamut-predicted impact on splice site selection of the proximal c.88-7T>A variant.

c.56-609G>C

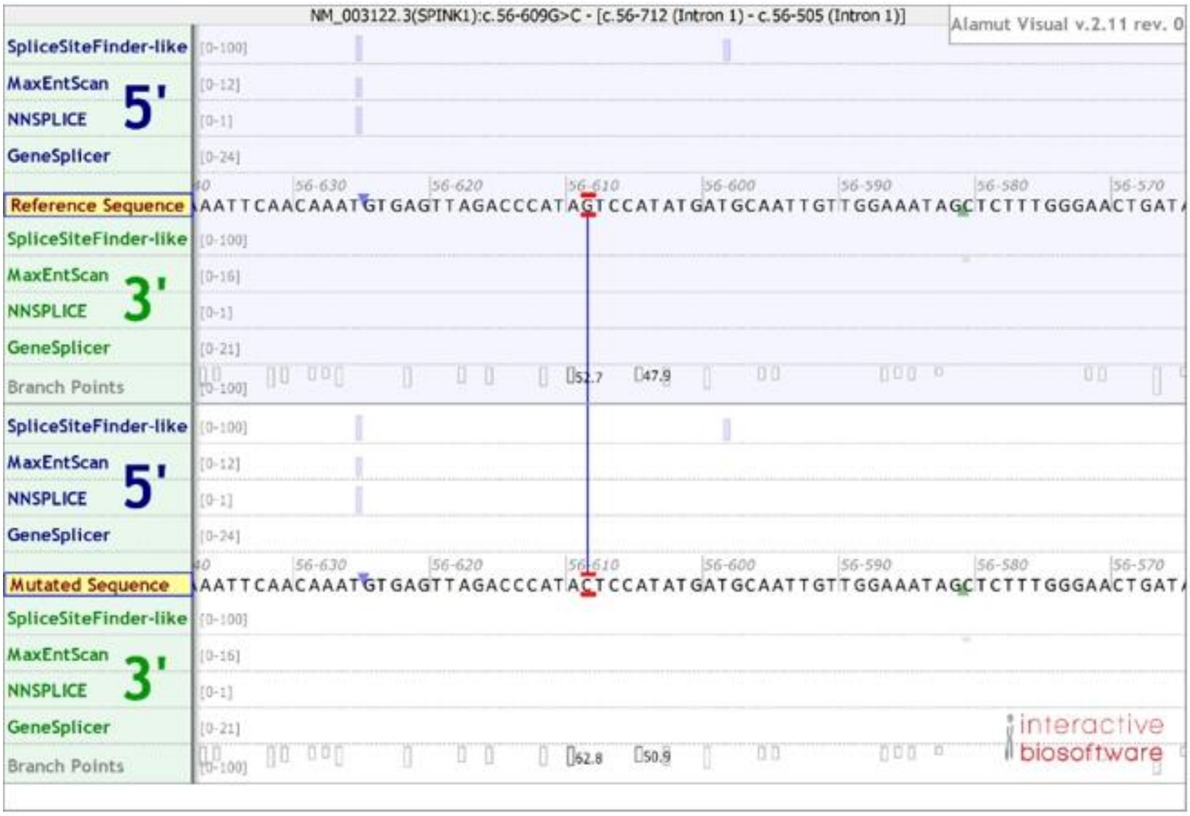

c.56-324T>A

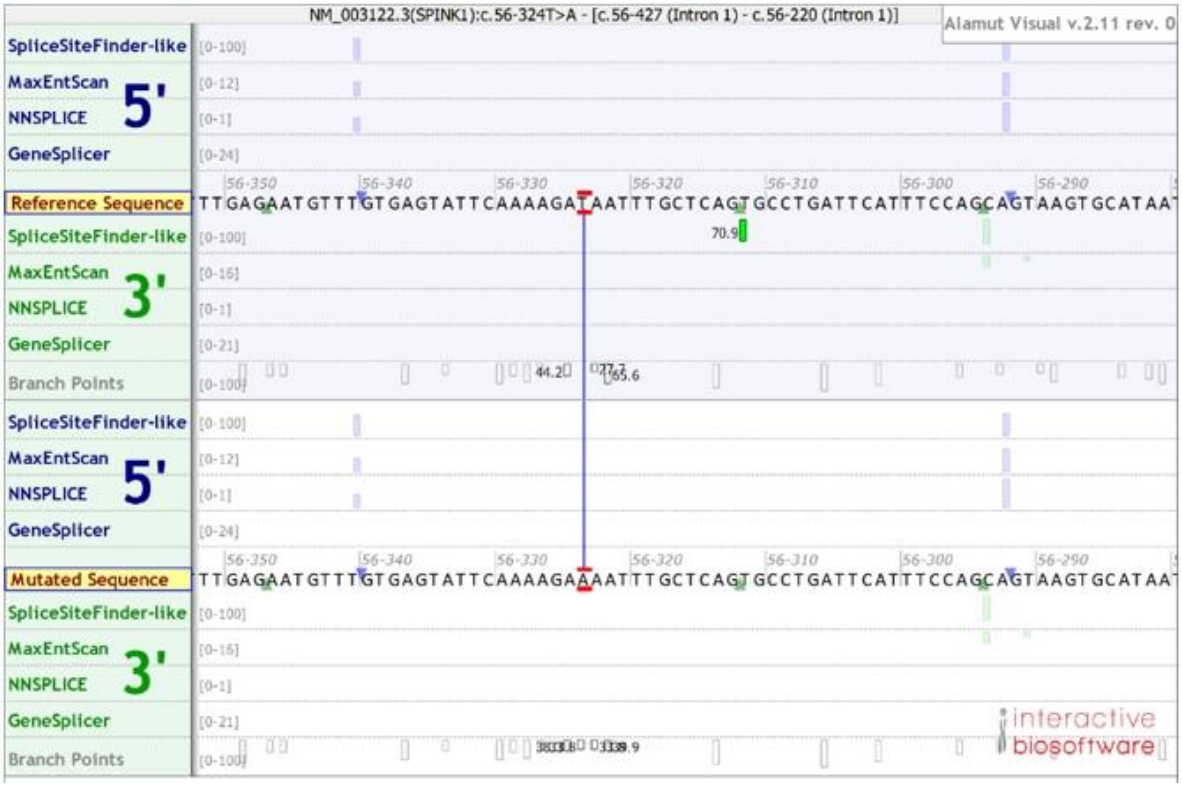

Figure S4. Alamut-predicted impact on splice site selection of the 10 deep *SPINK1* intronic variants with a minor allele frequency of <5% in the East Asian population.

c.194+671C>T

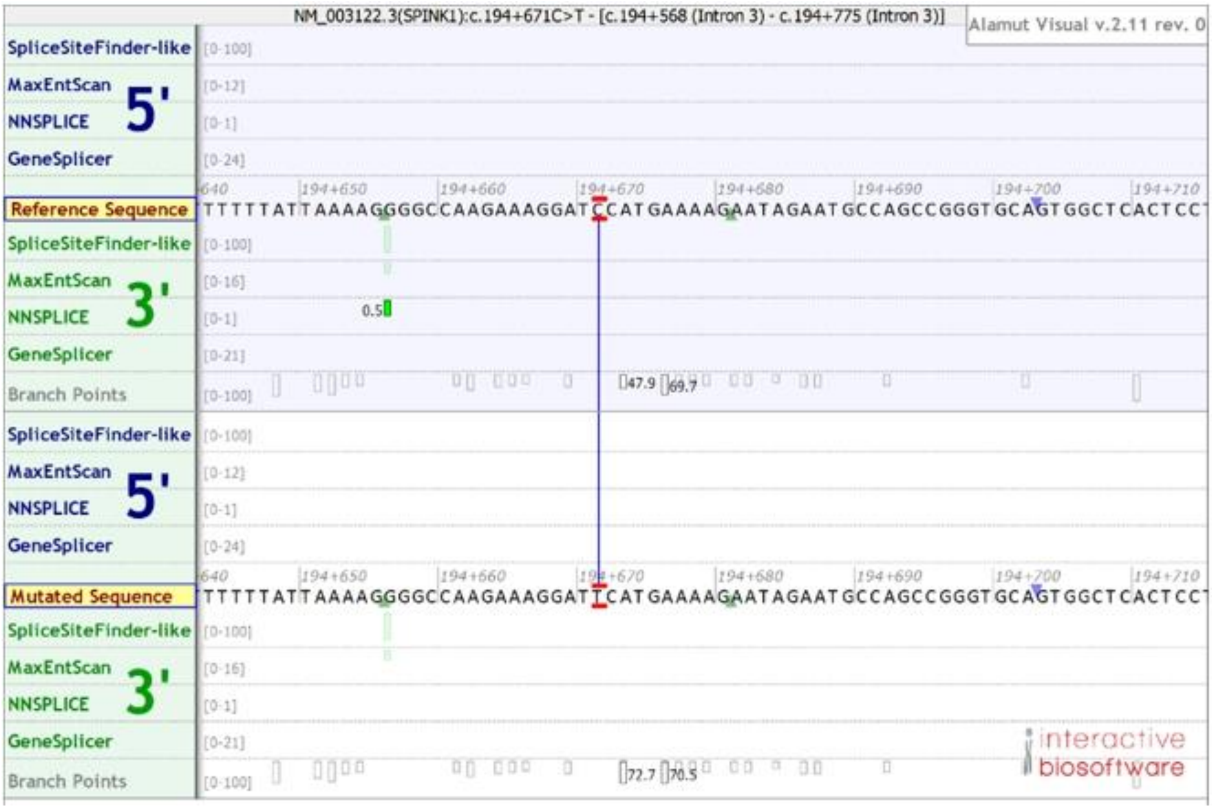

c.194+723C>T

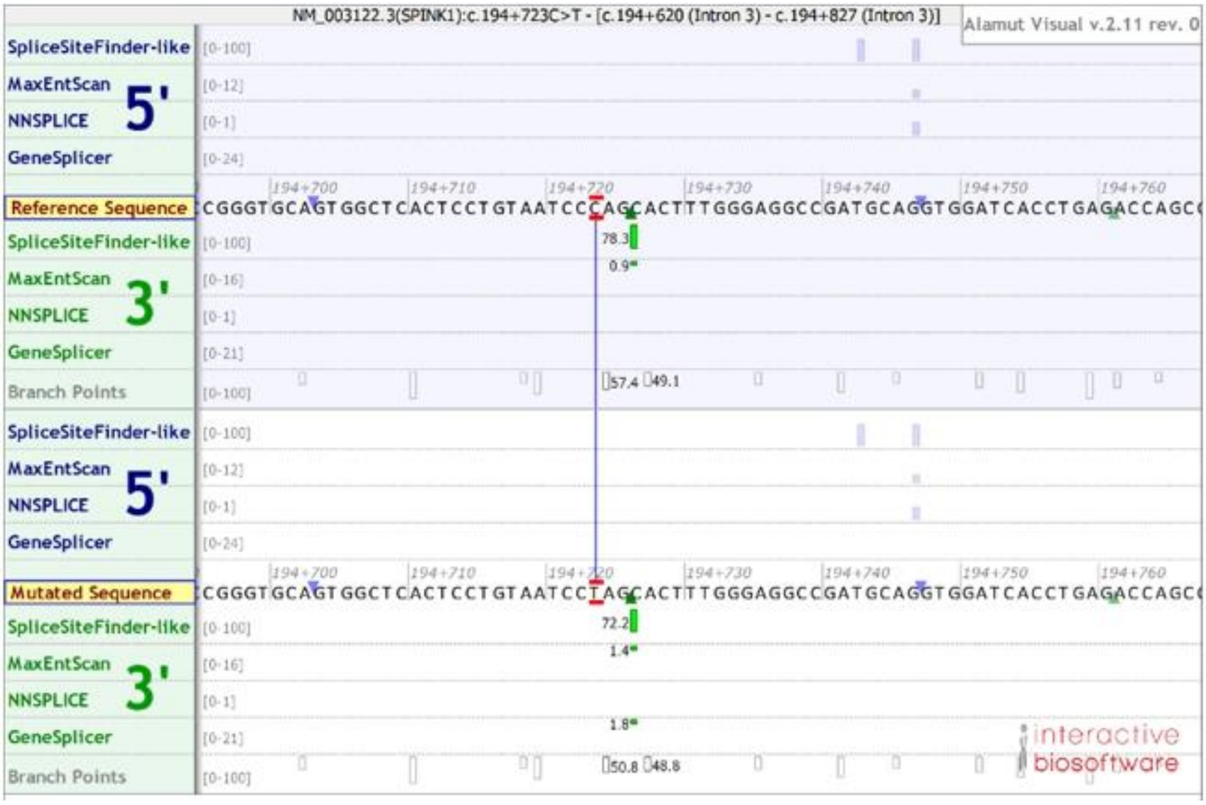

Figure S4 (continued)

c.194+855G>A

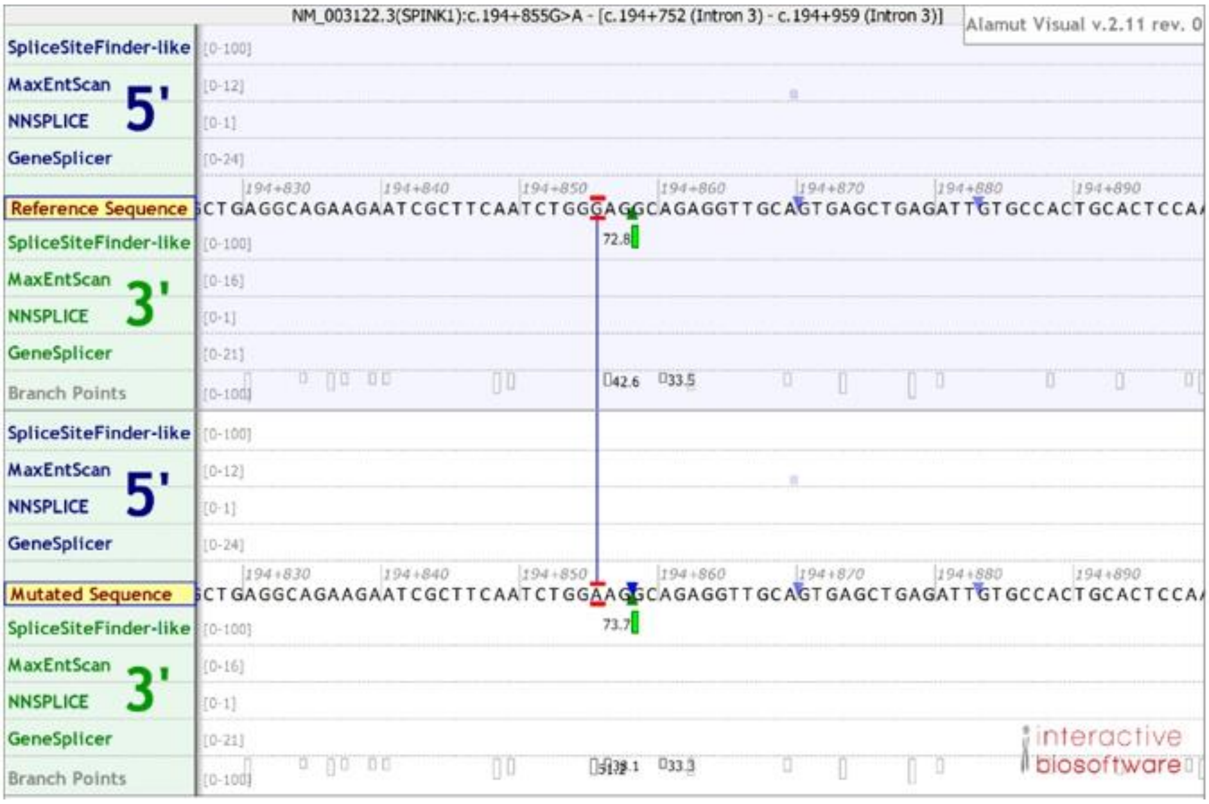

c.194+1278C>T

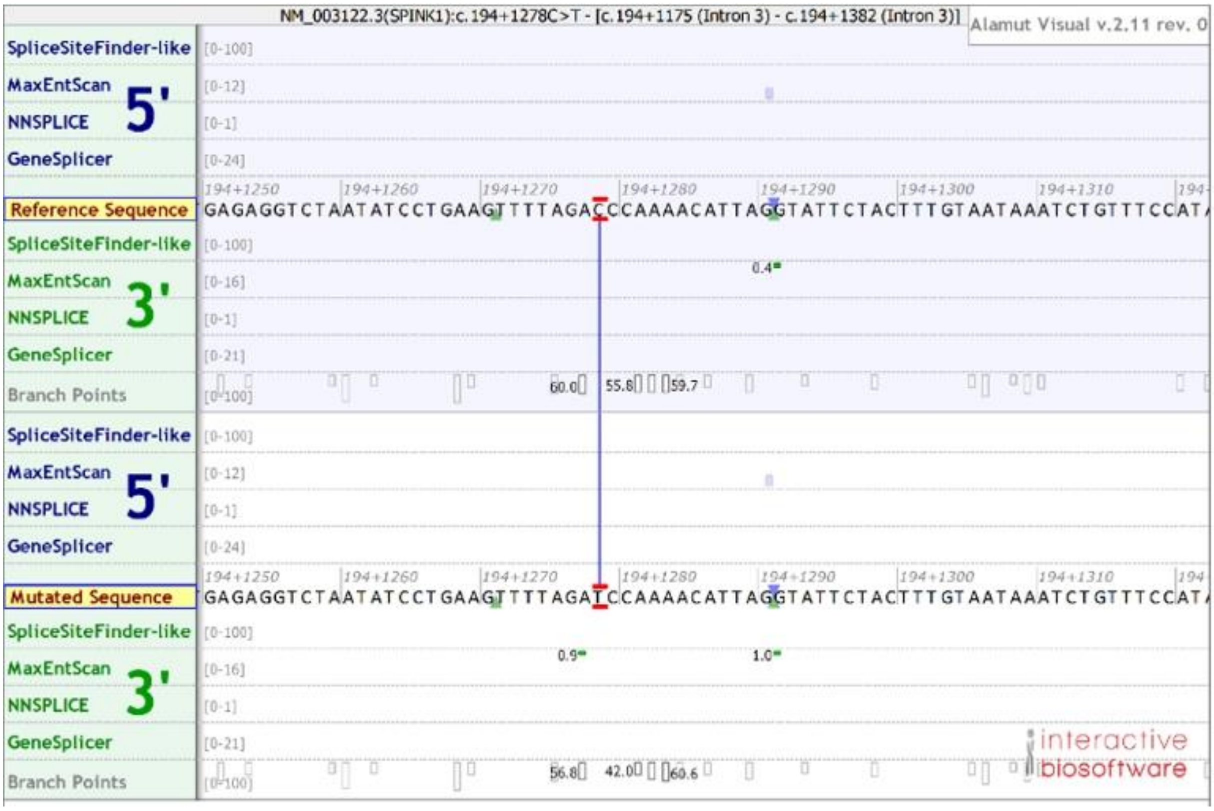

Figure S4 (continued)

c.194+1599G>A

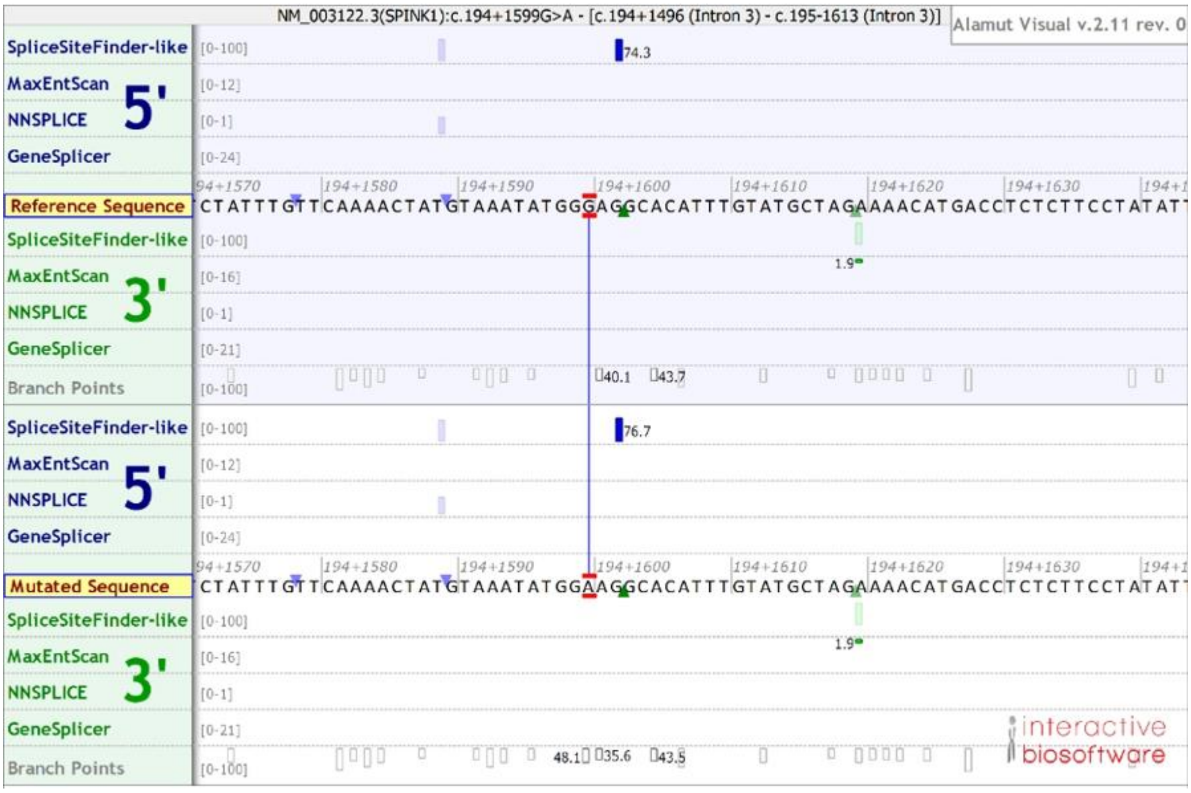

c.195-1414T>A (note that the minor allele sequence is used as the reference sequence)

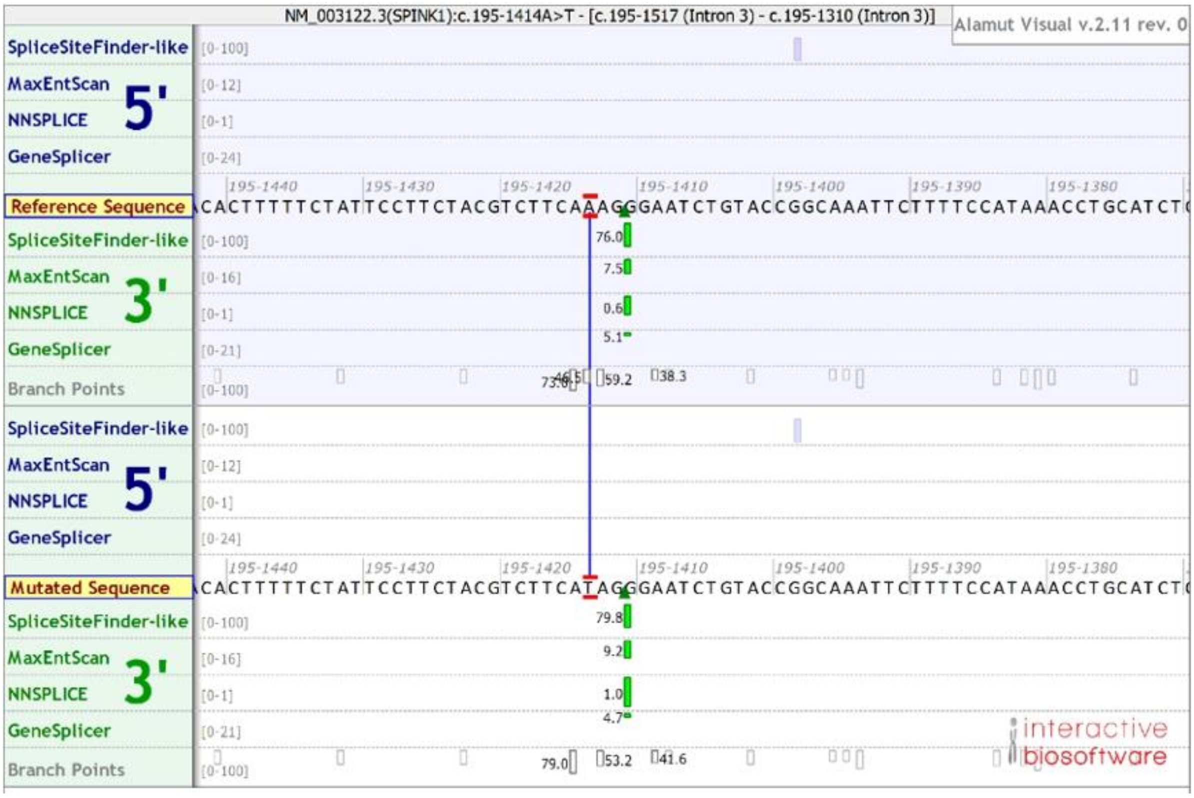

Figure S4 (continued)

c.195-862T>C

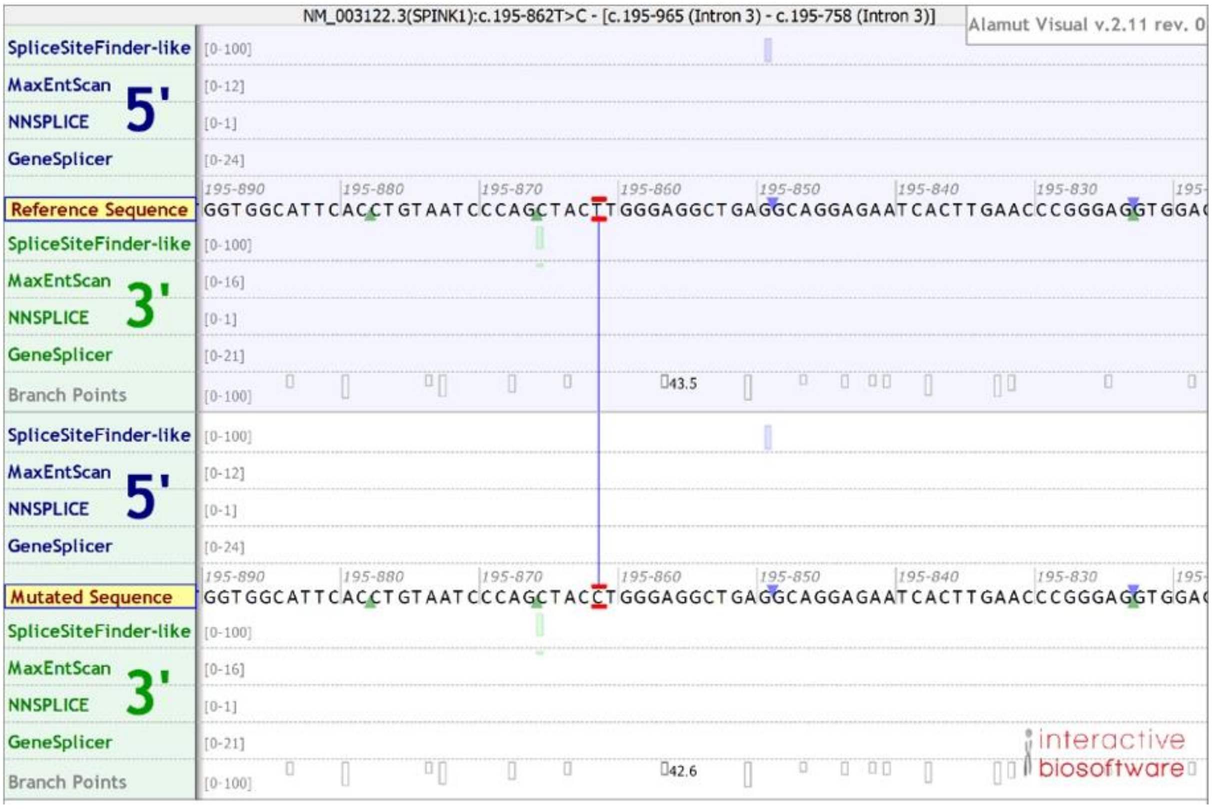

c.195-854C>T

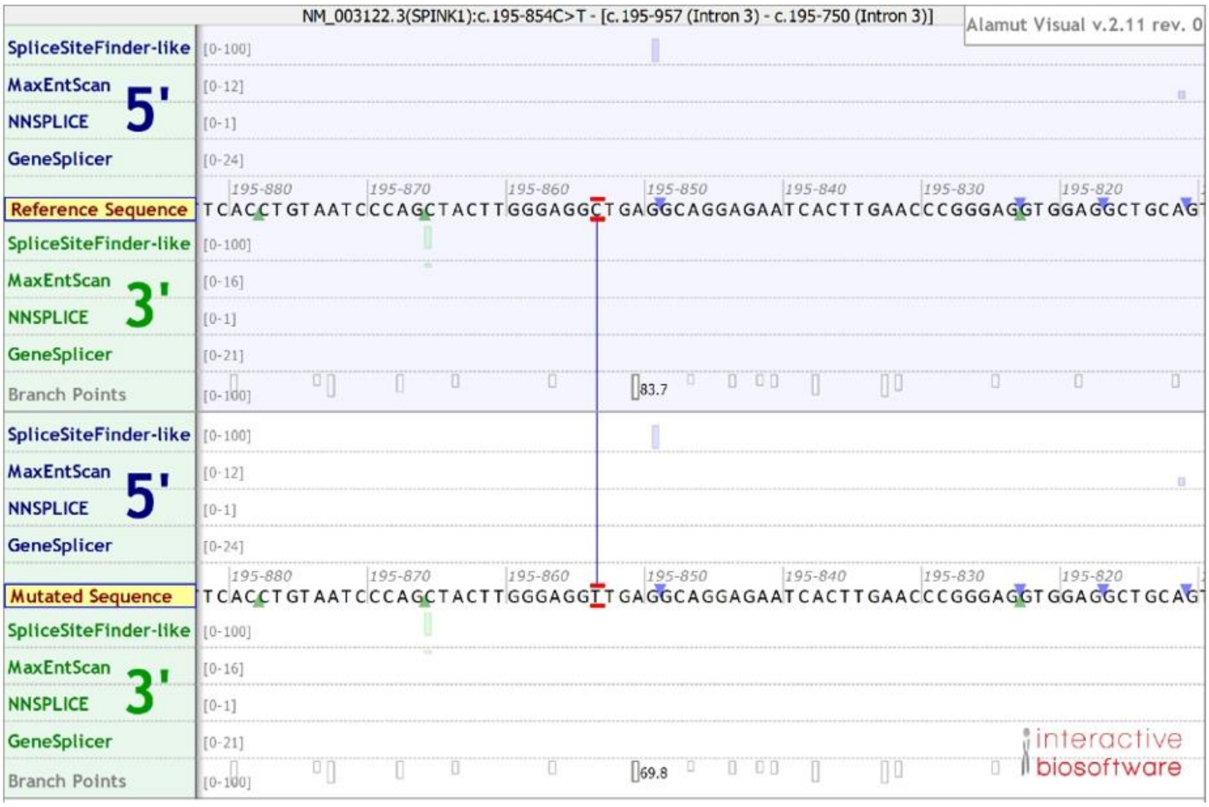

Figure S4 (continued)

c.87+13T>G

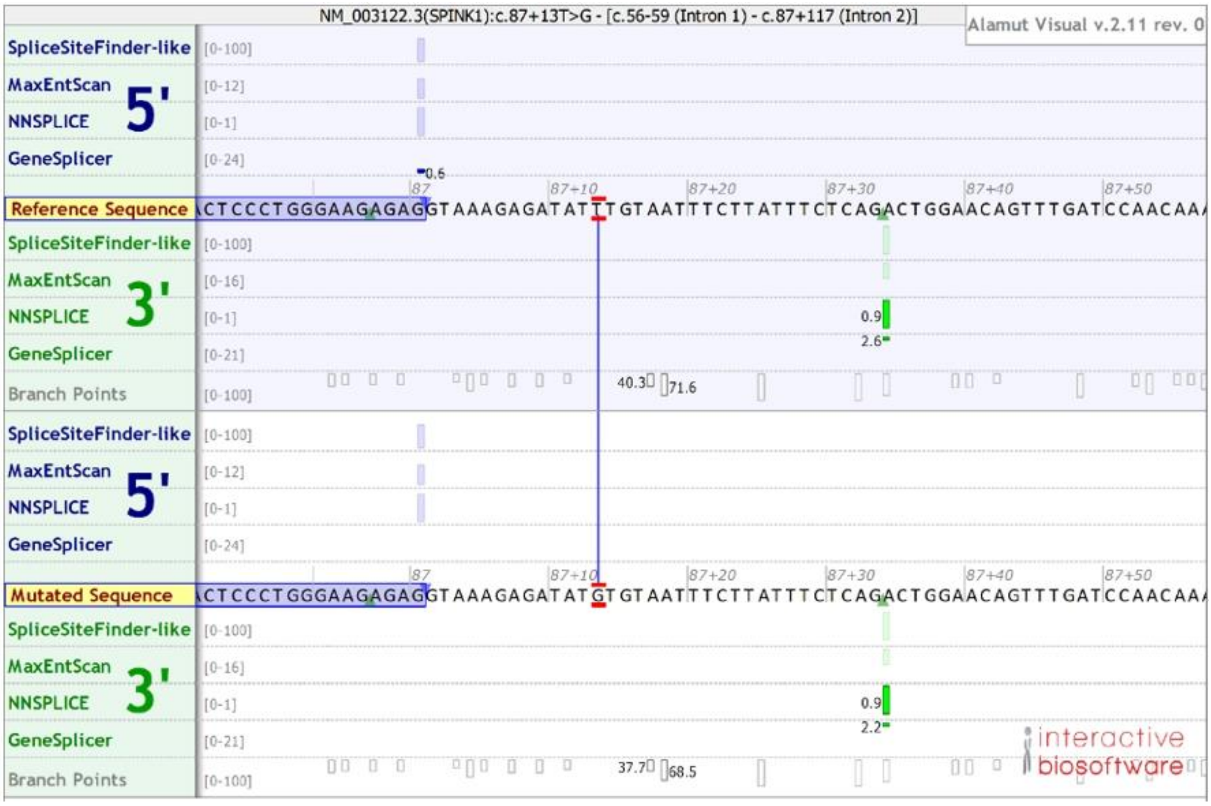

c.88-48C>A

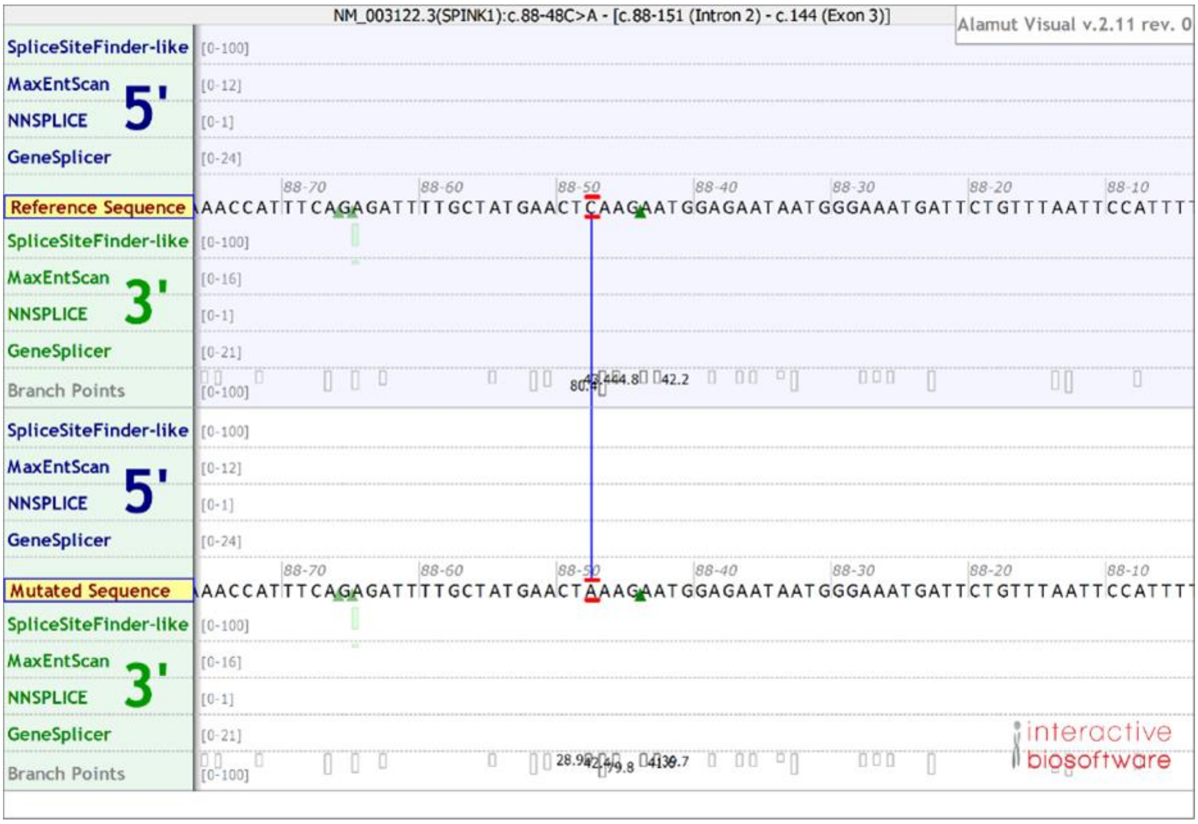

Figure S5. Alamut-predicted impact on splice site selection of the other four proximal *SPINK1* intronic variants found in the French pancreatitis patients.

c.194+32T>C

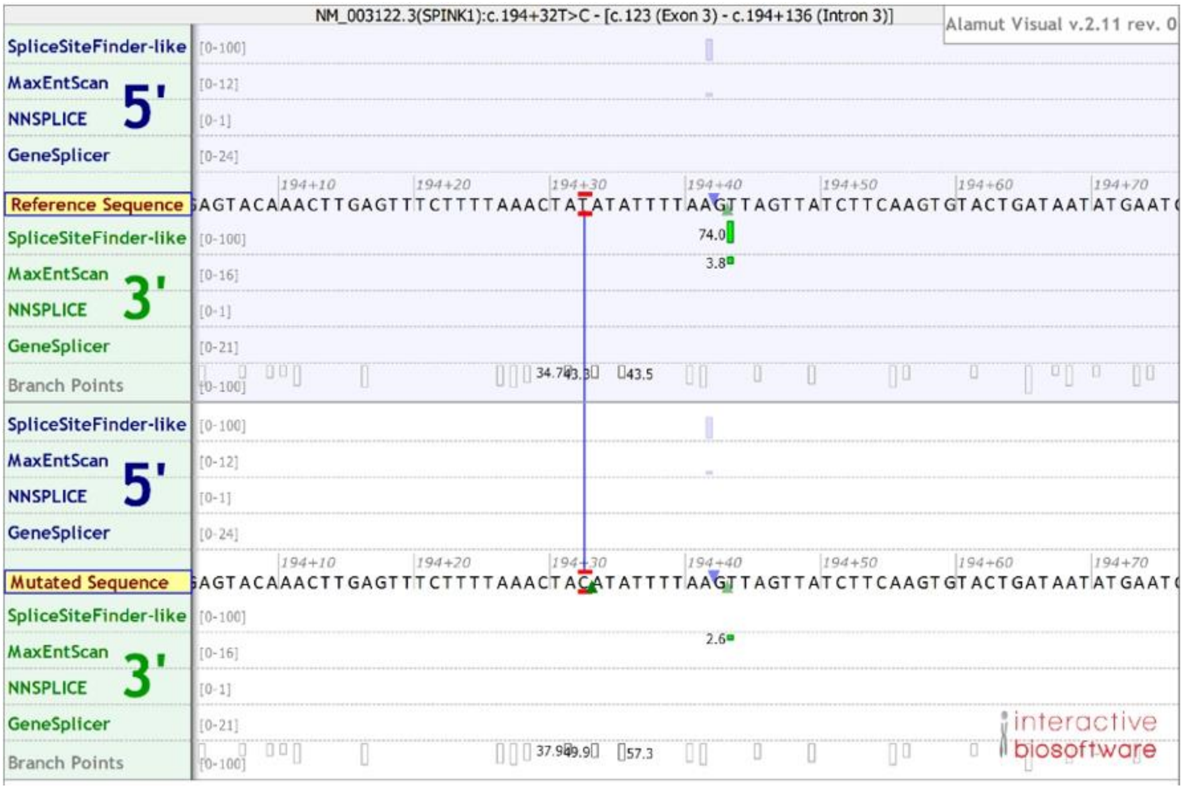

c.195-21T>A

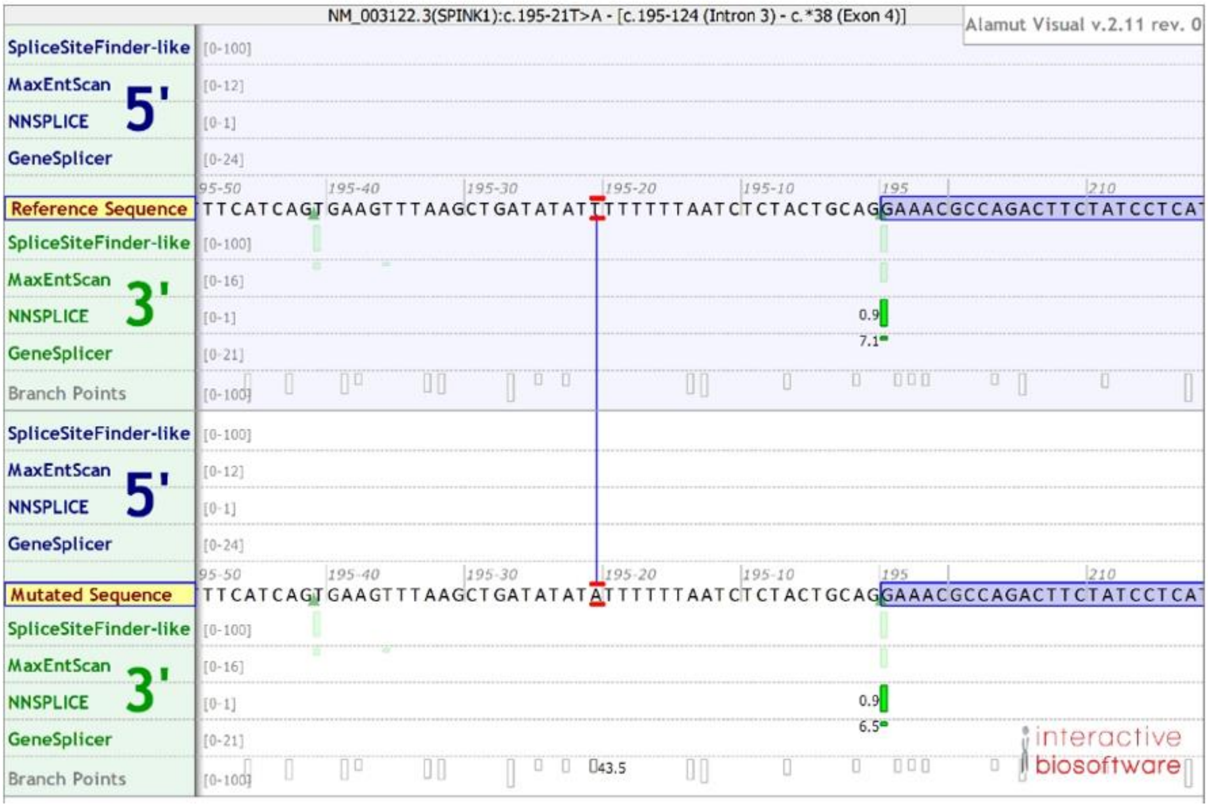

Figure S5 (continued)

c.88-352A>G

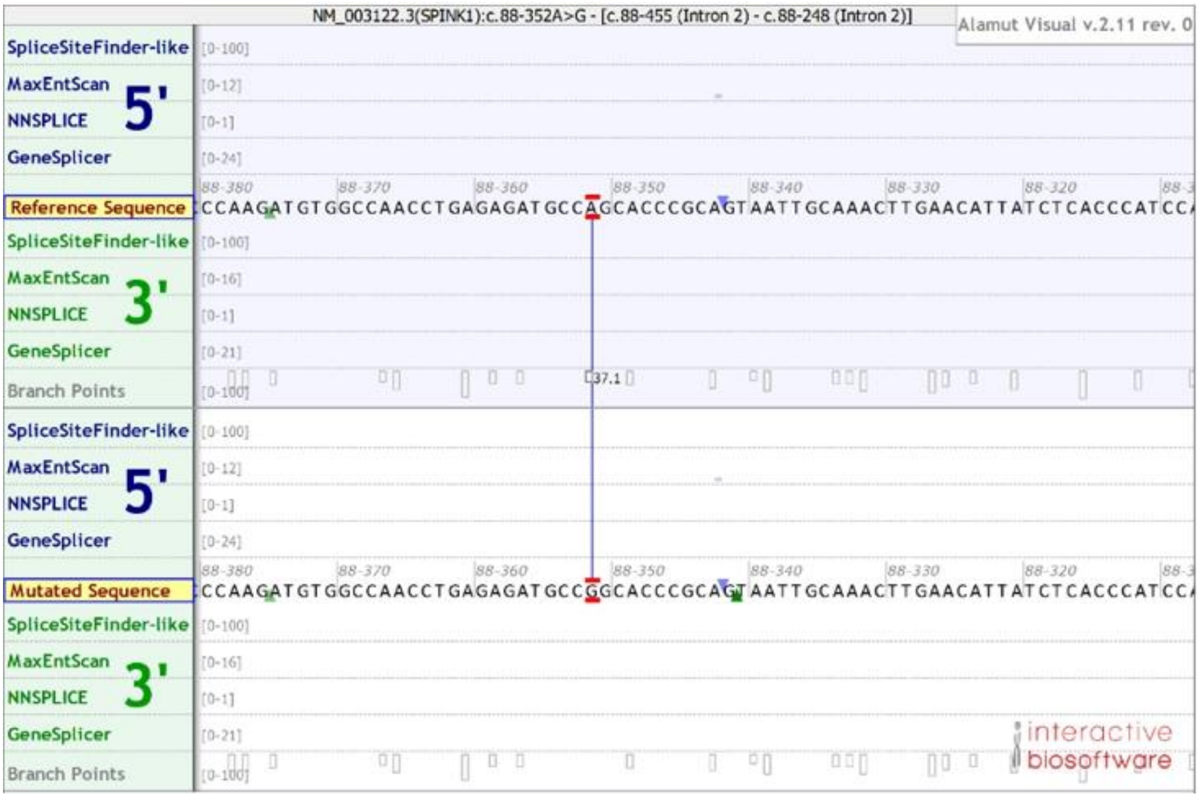

c.194+1159C>G

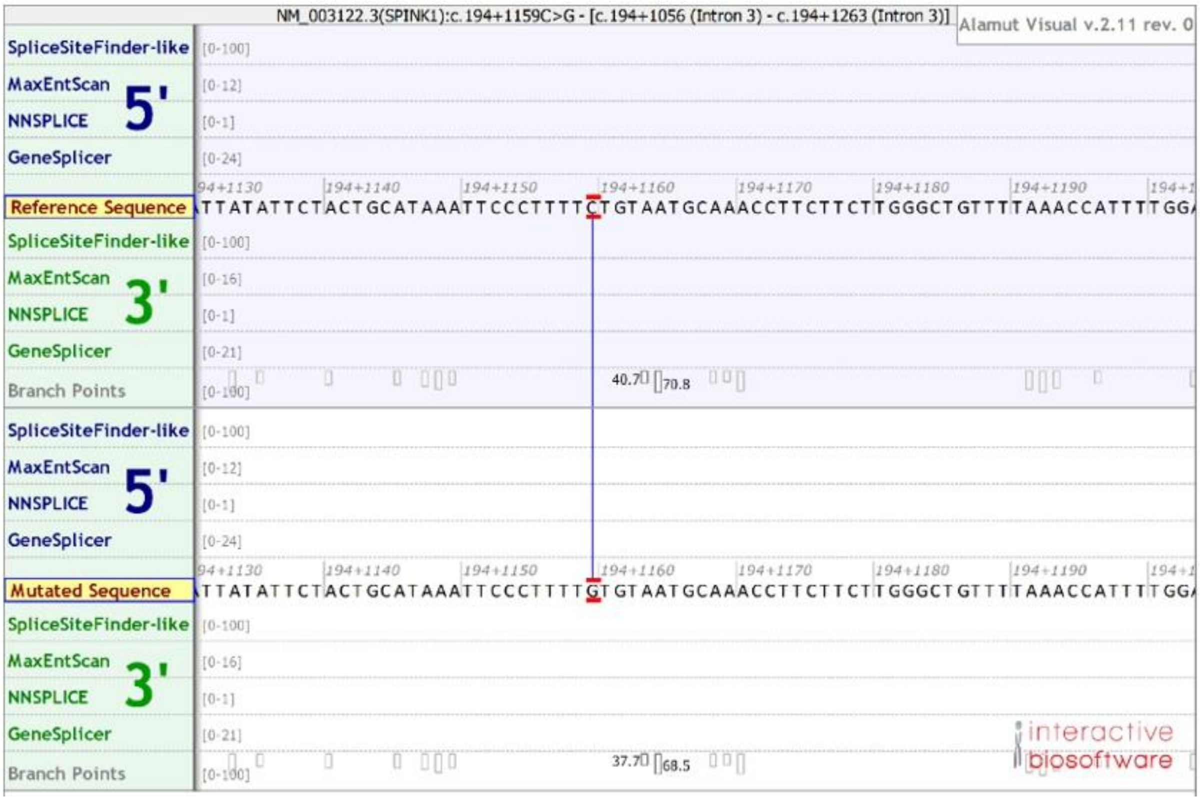

Figure S6. Alamut-predicted impact on splice site selection of the six deep *SPINK1* intronic variants with a minor allele frequency of ≥5% in the East Asian population.

c.195-1645G>C

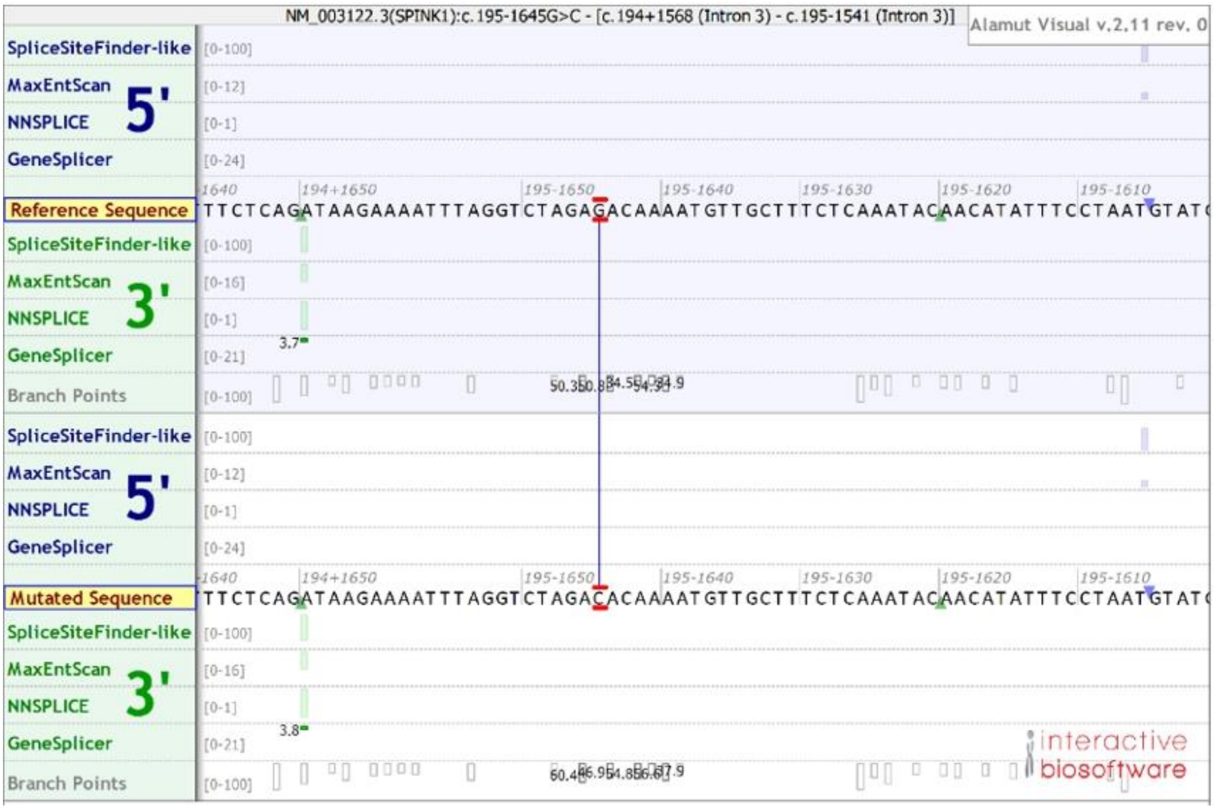

c.195-1570C>A

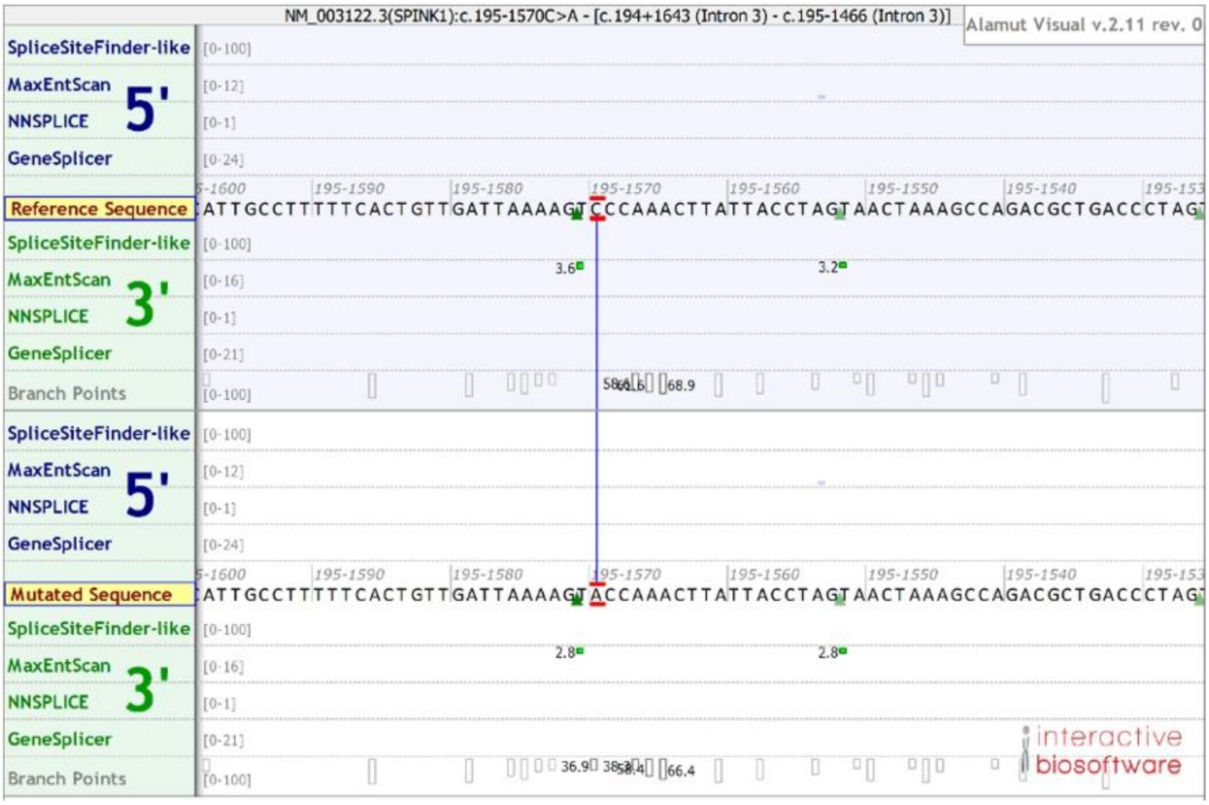

Figure S6 (continued)

c.195-478T>G

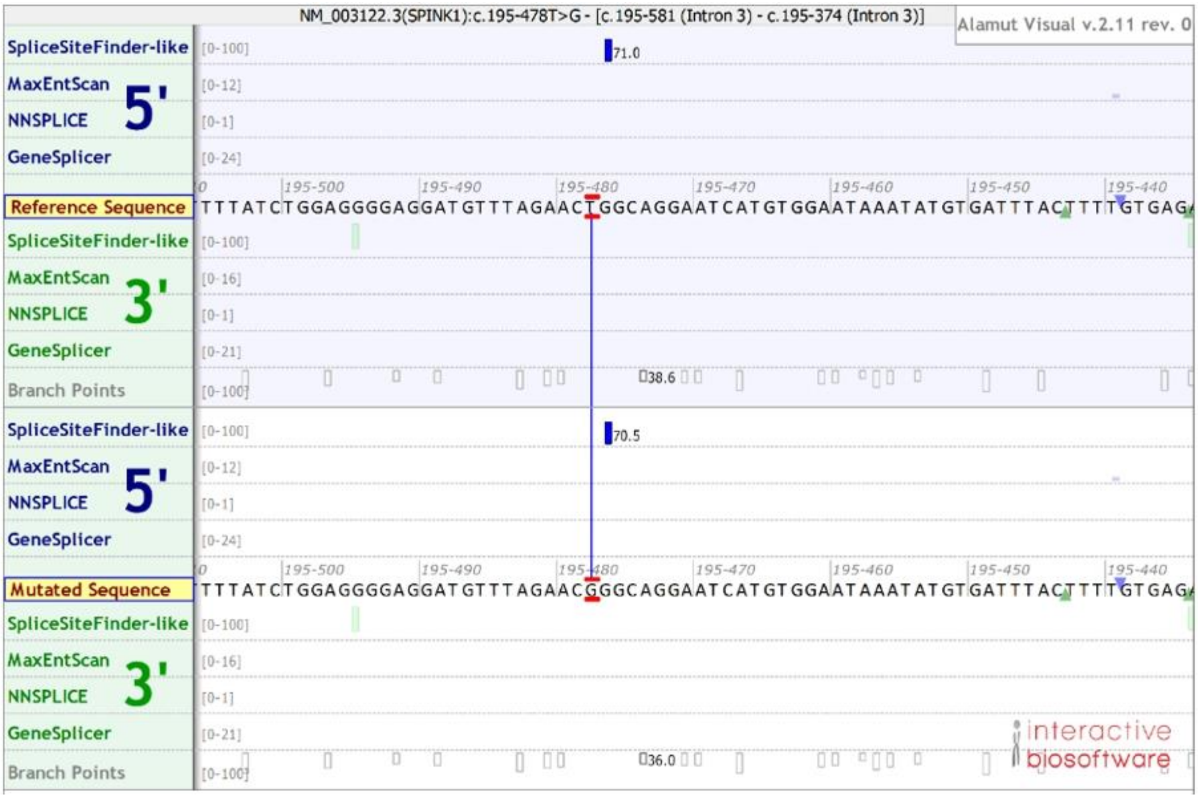

c.195-323C>T

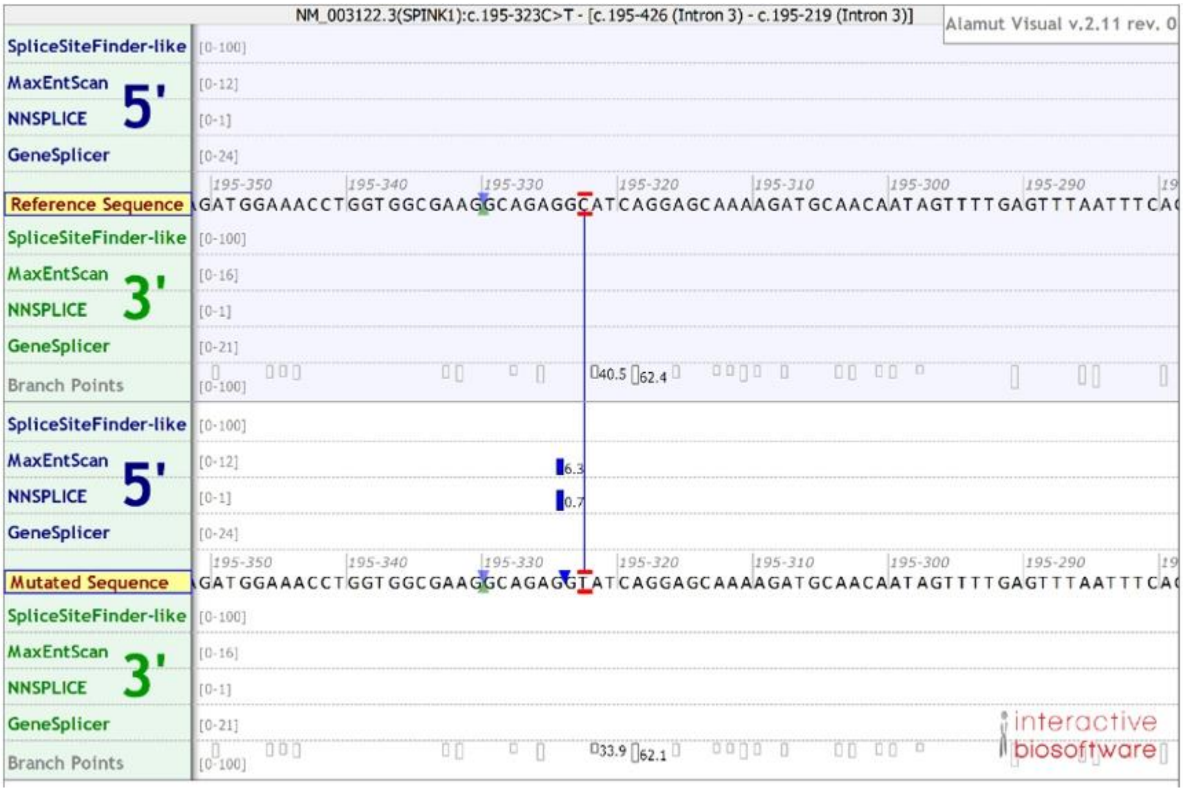

Figure S6 (continued)
